# *CUL4B* mutations impair human cortical neurogenesis through PP2A-dependent inhibition of AKT and ERK

**DOI:** 10.1038/s41419-024-06501-3

**Published:** 2024-02-08

**Authors:** Yanyan Ma, Xiaolin Liu, Min Zhou, Wenjie Sun, Baichun Jiang, Qiao Liu, Molin Wang, Yongxin Zou, Qiji Liu, Yaoqin Gong, Gongping Sun

**Affiliations:** 1 Key Laboratory of Experimental Teratology, Ministry of Education, Institute of Molecular Medicine and Genetics, School of Basic Medical Sciences, Cheeloo College of Medicine, Shandong University, Jinan, Shandong 250012 China; 2 Key Laboratory of Experimental Teratology, Ministry of Education, Department of Histology and Embryology, School of Basic Medical Sciences, Cheeloo College of Medicine, Shandong University, Jinan, Shandong 250012 China

**Keywords:** Developmental neurogenesis, Mechanisms of disease

## Abstract

Mutation in *CUL4B* gene is one of the most common causes for X-linked intellectual disability (XLID). CUL4B is the scaffold protein in CUL4B-RING ubiquitin ligase (CRL4B) complex. While the roles of CUL4B in cancer progression and some developmental processes like adipogenesis, osteogenesis, and spermatogenesis have been studied, the mechanisms underlying the neurological disorders in patients with *CUL4B* mutations are poorly understood. Here, using 2D neuronal culture and cerebral organoids generated from the patient-derived induced pluripotent stem cells and their isogenic controls, we demonstrate that CUL4B is required to prevent premature cell cycle exit and precocious neuronal differentiation of neural progenitor cells. Moreover, loss-of-function mutations of *CUL4B* lead to increased synapse formation and enhanced neuronal excitability. Mechanistically, CRL4B complex represses transcription of *PPP2R2B* and *PPP2R2C* genes, which encode two isoforms of the regulatory subunit of protein phosphatase 2 A (PP2A) complex, through catalyzing monoubiquitination of H2AK119 in their promoter regions. *CUL4B* mutations result in upregulated PP2A activity, which causes inhibition of AKT and ERK, leading to premature cell cycle exit. Activation of AKT and ERK or inhibition of PP2A activity in *CUL4B* mutant organoids rescues the neurogenesis defect. Our work unveils an essential role of CUL4B in human cortical development.

## Introduction

Intellectual disability is a large group of neurological disorders characterized by significant limitations in intellectual and adaptive skills [1]. X-linked intellectual disability (XLID) accounts for about 10% of all inherited cases of intellectual disability in males and occurs at a frequency of 1 in 600 males [2]. Previously, our group and others have shown that mutations in *CUL4B* result in XLID [3, 4]. So far, *CUL4B* mutations have been found in 3% of XLID cases [5, 6]. Patients with *CUL4B* mutations exhibit severe intellectual disability, aphasia, seizure, increased monocytes, central obesity and short stature [7]. Neuroimaging data of some patients reveal cerebral abnormalities [8], implying that CUL4B is essential for cerebral development.

CUL4B serves as a scaffold protein in CUL4B-RING E3 ligase (CRL4B) complex. Studies over the past two decades have demonstrated that CRL4B complex can regulate myeloid functions, adipogenesis and osteogenesis [9–11], providing insights for mechanisms underlying some phenotypes in patients with *CUL4B* mutations. In addition, CUL4B has been intensively reported to promote growth, metastasis and drug resistance in many solid tumors [12–14]. However, the mechanisms underlying the neurological defects caused by *CUL4B* mutation are far from clear. Deletion of *Cul4b* in mice leads to reduced PV-positive neurons and alters dendritic features in the hippocampus, resulting in increased epileptic susceptibility and spatial learning defects [15]. Mice lacking CUL4B in nervous system display increased abundance of GFAP-positive cells through upregulation of PTGDS [16]. However, mouse models cannot fully recapitulate the defects in human central nervous system due to differences in brain complexity between species.

Neuronal cells and brain organoids derived from human induced pluripotent stem cells (iPSCs) are powerful tools in studying mechanisms underlying human diseases in patient-specific genetic backgrounds [17–19]. In this study, we generated cerebral organoids using iPSCs derived from two XLID patients with loss-of-function mutations of *CUL4B*. By comparing to their isogenic controls, we demonstrate that *CUL4B* deficiency results in premature cell cycle exit and precocious neuronal differentiation of neural progenitor cells (NPCs), unbalanced production of deep-layer and upper-layer neurons, increased synapse formation and neuronal hyperexcitability in cerebral organoids. Mechanistically, loss of CUL4B results in de-repression of *PPP2R2B* and *PPP2R2C*, leading to PP2A-mediated inhibition of AKT and ERK activities. Inhibition of PP2A or activation of AKT and ERK rescues the neurogenesis defects in *CUL4B*-deficient cerebral organoids.

## Results

### Lack of CUL4B causes premature cell cycle exit and precocious neuronal differentiation of NPCs

**Fig. 1:**
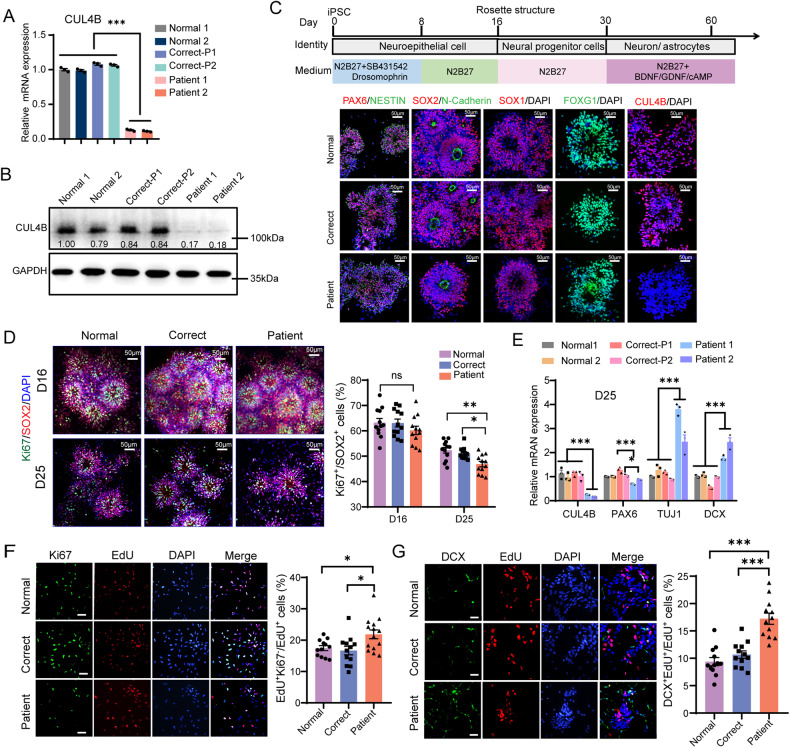
*CUL4B* deficiency results in premature cell cycle exit and precocious neuron differentiation of NPCs in 2D culture. **A** The mRNA levels of *CUL4B* in different iPSC lines were quantified by qRT-PCR. N = 3. **B** Western blots showing CUL4B protein levels in different iPSC lines. The number below each CUL4B band is the normalized ratio between the intensity of CUL4B band and the intensity of GAPDH band from the same sample. **C** The upper is the schematic showing neural differentiation strategy for iPSCs. The lower is the immunostaining for the NPC markers PAX6, NESTIN, SOX2, N-Cadherin, SOX1, the forebrain marker FOXG1 and CUL4B in NPCs on day 17. Scale bar, 50 μm. **D** The representative images of NPCs stained with SOX2 and Ki67 in day-16 (D16) and day-25 (D25) cell cultures and quantifications of the percentage of Ki67^+^ cells among SOX2^+^ cells. Scale bar, 50 μm. N = 12. **E** The mRNA levels of *CUL4B*, *PAX6*, *TUJ1* and *DCX* in different cell lines. N = 3. **F** Ki67 staining (green) of NPCs at 48 h after EdU labeling (red). Scale bar, 50μm. The bar graph shows the percentage of EdU^+^ Ki67^−^ cells in all EdU^+^ cells. N = 12-14. **G** DCX staining (green) of NPCs at 48 h after EdU labeling (red). Scale bar, 50 μm. The bar graph shows the percentage of EdU^+^ DCX^+^ cells in all EdU^+^ cells. N = 12. The statistical significance was determined using one-way ANOVA with Tukey test. *: *P* < 0.05; **: *P* < 0.01; ***: *P* < 0.001. ns: no significance.

To address the role of CUL4B in neurodevelopment, we employed two iPSC lines previously generated by our group from peripheral blood mononuclear cells (PBMCs) of two independent XLID patients (SDUBMSI002-A cell line and SDQLCHi015-A cell line) [20, 21]. SDUBMSI002-A cell line, here named as Patient 1, carries a non-sense *CUL4B* c.1564 C > T mutation. SDQLCHi015-A cell line, annotated as Patient 2 in this study, harbors *CUL4B* c.1007_1011del mutation that causes a frameshift and premature translational termination. In addition, we generated four control iPSC lines. Two of them were derived from PBMCs from two age- and sex-matched healthy donors (designated as Normal 1 and Normal 2, respectively; Supplementary Table S1). The other two lines were the isogenic controls for Patient 1 and Patient 2 lines by correcting the mutations using CRISPR-Cas9 gene editing (designated as Correct-P1 and Correct-P2, respectively; Supplementary Table S1, Supplementary Figure S1A-F). Both Correct-P1 and Correct-P2 iPSCs had CUL4B expression similar to Normal 1 and Normal 2 iPSCs, whereas no CUL4B protein was detected in the patient-derived iPSCs (Fig. 1A, B). All the iPSC lines displayed similar expression of pluripotency markers (Supplementary Figure S1G), suggesting that *CUL4B* deficiency does not affect the pluripotency.

We employed a modified 2-dimensional (2D) differentiation protocol to generate forebrain-specific cortical neurons from the patient-derived and the control iPSCs (Fig. 1C, Supplementary Figure S2A). All the cell lines lost expression of pluripotency genes OCT4 and NANOG by day 8 of differentiation and expressed similar levels of NPC markers by day 16 of differentiation (Fig. 1C, Supplementary Figure S2B, C), suggesting that loss of CUL4B does not influence differentiation of iPSCs into NPCs. On day 16, both the patient-derived and the control NPCs displayed similar proliferation. However, a significant reduction in the percentage of Ki67^+^ cells was observed in the patient-derived cells compared to the Normal controls and the isogenic controls on day 25 (Fig. 1D), when NPCs started to differentiate into neurons [22] (Supplementary Figure S2A). The reduction was not due to elevated cell death (Supplementary Figure S2D). In the meanwhile, increased expression of neuron markers doublecortin (DCX) and β-tubulin III (TUJ1) was detected in the patient-derived cells compared to the Normal and the isogenic controls on day 25 (Fig. 1E). These data suggest an involvement of CUL4B in regulating the switch from proliferation to differentiation in NPCs. To clarify the role of CUL4B in cell cycle exit and neuronal differentiation in NPCs, we pulse labeled the NPCs with EdU and stained for Ki67 or DCX at 48 h post-labeling. The patient-derived lines exhibited elevated percentage of Ki67^-^ EdU^+^ cells and DCX^+^ EdU^+^ cells among EdU^+^ cells compared to the Normal and the isogenic controls (Fig. 1F, G), indicating increased cell cycle exit and neuronal differentiation in the NPCs lacking CUL4B. These data together suggest that CUL4B is essential for preventing premature cell cycle exit and precocious differentiation of NPCs.

**Fig. 2:**
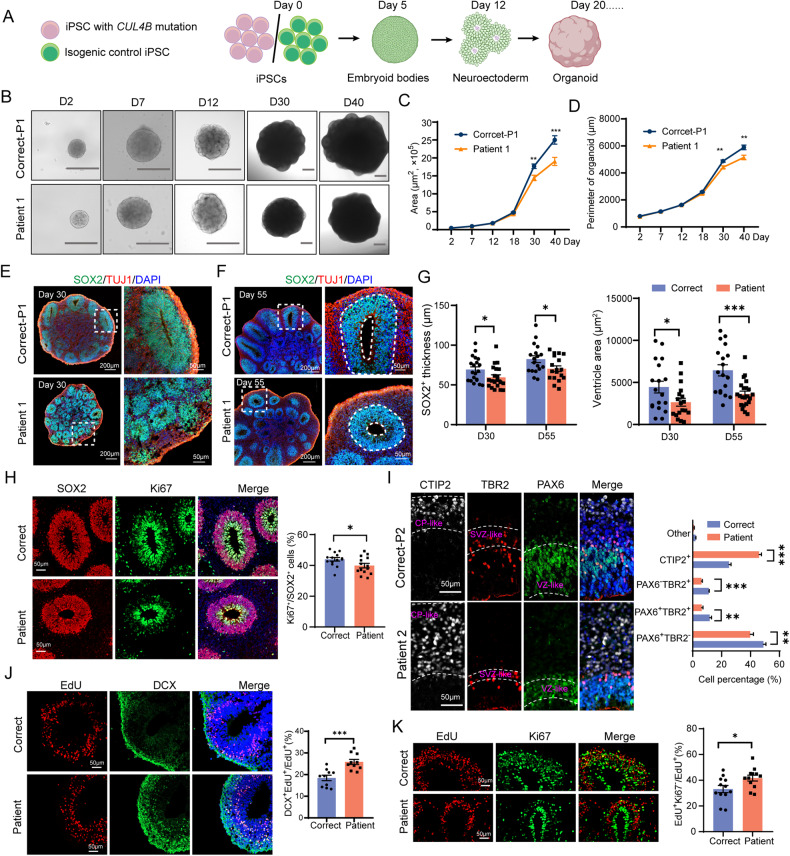
The patient-derived cerebral organoids display premature cell cycle exit and precocious neuronal differentiation of NPCs. **A** The schematic of generation of cerebral organoids from iPSCs. **B** Bright-field images of the patient-derived cerebral organoids (Patient 1) and the isogenic control organoids (Correct-P1) at different time points. D, day. Scale bar, 400 μm. Quantifications of the area (**C**) and the perimeter (**D**) of organoids at different time points. More than 15 organoids from three independent experiments were analyzed for each line. Representative images of SOX2 and TUJ1 staining of day-30 (**E**) and day-55 (**F**) organoids. Scale bar, 200 μm in the left column and 50 μm in the right column. **G** Quantification of the thickness of SOX2^+^ layers and ventricle area in organoids. 18 to 24 images from four individual organoids from each group were counted. **H** Representative images of SOX2 and Ki67 staining on day-55 organoids. Scale bar, 50 μm. The bar graph shows the percentage of Ki67^+^ cells in SOX2^+^ cells. 14 to 18 images from four individual organoids from each group were counted. **I** Representative images of the isogenic control (Correct) and the patient-derived (Patient) cortical organoids immunostained with CTIP2, TBR2 and PAX6 antibodies. Scale bar, 50 μm. The inferred VZ-, SVZ-, and CP-like regions are delineated based on the combination of DAPI, PAX6, TBR2, and CTIP2 staining patterns. The bar graph shows the percentages of the indicated cells in Correct (12 total organoids from 2 isogenic control lines) and Patient (12 organoids from 2 patient-derived lines) organoids. **J** DCX staining (green) of day-28 organoids at 48 h after EdU labeling (red). Scale bar, 50 μm. The bar graph shows the percentage of EdU^+^ DCX^+^ cells in all EdU^+^ cells. N = 10 sections from five organoids each line. **K** Ki67 staining (green) of day-28 organoids at 48 h after EdU labeling (red). Scale bar, 50 μm. The bar graph shows the percentage of EdU^+^ Ki67^−^ cells in all EdU^+^ cells. N = 12 sections from six organoids each line. Data are presented as the mean ± SEM. The statistical significance was determined using two-tailed unpaired t-test. *: *P* < 0.05; **: *P* < 0.01; ***: *P* < 0.001. ns: no significance.

Next, we generated cerebral organoids from the patient-derived iPSCs (Patient 1 and Patient 2) and the isogenic controls (Correct-P1 and Correct-P2), using a previously described protocol [23](Fig. 2A). The growth of the patient-derived organoids was similar to that of their isogenic controls within the first 18 days but became slower thereafter. On day 30, the patient-derived organoids were significantly smaller than their isogenic controls. The difference in size was further exacerbated on day 40 (Fig. 2B-D, Supplementary Figure S3A-C). Both the ventricles and the SOX2^+^ NPC layers were smaller in the patient-derived organoids than in the isogenic controls (Fig. 2E–G, Supplementary Figure S3D-E). In addition, the patient-derived organoids displayed reduced proliferative SOX2^+^ NPCs compared to the isogenic controls (Fig. 2H). We then stained the organoids on day 60 with the NPC marker PAX6, the intermediate progenitor cell (IPC) marker TBR2 and the deep-layer cortical marker CTIP2. The PAX6^+^ TBR2^-^, the TBR2^+^ and the CTIP2^+^ regions resemble the developing ventricular zone (VZ), subventricular zone (SVZ) and the cortical plate (CP), respectively (Fig. 2I, Supplementary Figure S3F). We observed significantly reduced percentages of TBR2^+^ cells and PAX6^+^ cells and strongly increased percentage of CTIP2^+^ cells in the patient-derived organoids compared to the isogenic controls (Fig. 2I), suggesting depletion of progenitor cells and elevated neuron differentiation. The EdU pulse labeling assays in the organoids also revealed increased cell cycle exit and neuron differentiation in *CUL4B*-deficient NPCs (Fig. 2J, K). Data from both 2D neural culture and cerebral organoids indicate that CUL4B is essential for preventing premature switch from proliferation to differentiation in NPCs.

### *CUL4B* deficiency results in unbalanced differentiation into deep-layer and upper-layer neurons

**Fig. 3:**
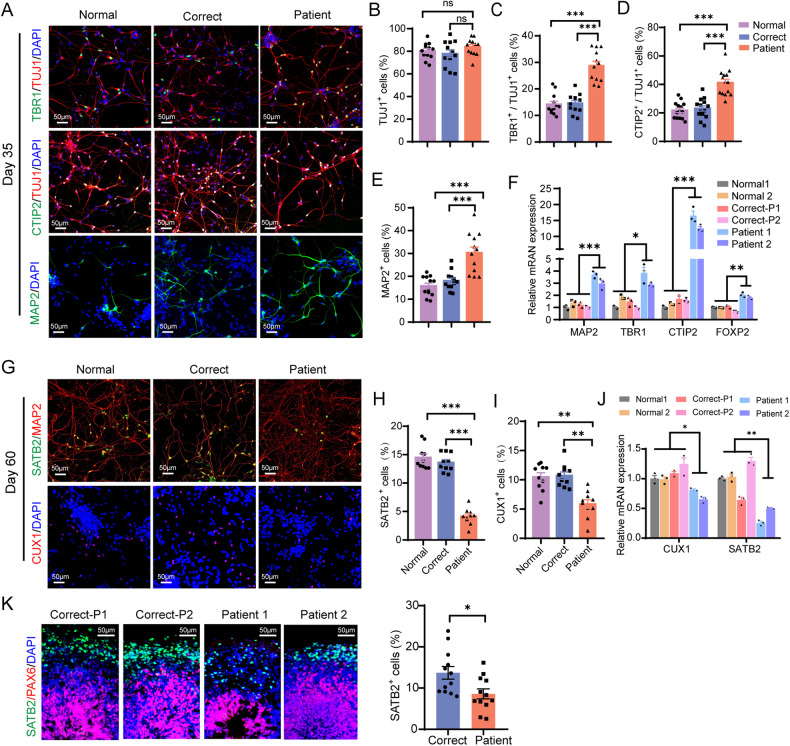
Lack of CUL4B results in unbalanced differentiation into deep-layer and upper-layer neurons. **A** Immunostaining of TUJ1, MAP2, TBR1 and CTIP2 on day 35 of neural differentiation. Scale bar, 50 μm. **B** Quantification of the percentage of TUJ1^+^ cells in all cells. N = 12. Quantifications of the percentage of TBR1^+^ cells (**C**) or CTIP2^+^ cells (**D**) in TUJ1^+^ cells. N = 12-14. **E** Quantification of the percentage of MAP2^+^ cells in all cells. N = 12. **F** mRNA levels of *MAP2*, *TBR1*, *CTIP2* and *FOXP2* on day 35. **G** Immunostaining of MAP2, SATB2, CUX1 on day 60 of neural differentiation. Scale bar, 50 μm. Quantifications of SATB2^+^ cells (**H**) and CUX1^+^ cells (**I**). N = 10. **J** mRNA levels of *CUX1*, *SATB2* on day 60. **K** Representative images of day 60 organoids stained with antibodies against PAX6 and SATB2. Scale bar, 50μm. The bar graph shows the proportion of SATB2^+^ cells in the isogenic control (Correct) and the patient-derived (Patient) organoids. N = 12. Data are presented as the mean ± SEM. The statistical significance was determined using one-way ANOVA with Tukey test for comparing three or more samples and two-tailed unpaired t-test for two-sample comparison. *: *P* < 0.05; **: *P* < 0.01; ***: *P* < 0.001. ns: no significance.

During neocortex development in human, NPCs generate different cell types through a fixed temporal order, with the deepest neuronal layers emerging first and the upper-layer neurons appearing later [24]. We evaluated generation of the deep-layer and the upper-layer neurons during neuronal differentiation in 2D culture. On day 35 of differentiation, all cell lines had the majority of cells (>75%) differentiated into TUJ1^+^ neurons (Fig. 3A, B). Immunostaining for the deep-layer neuron markers, TBR1 and CTIP2, revealed a dramatic increase in the percentage of the deep-layer cortical neurons in the patient-derived neurons compared to that in the Normal and isogenic control neurons (Fig. 3A, C, D). The percentage of MAP2^+^ mature neurons was also elevated in the patient-derived cells on day 35 compared to the other two groups (Fig. 3A, E). Consistently, the mRNA levels of the deep-layer neuron marker genes *TBR1*, *CTIP2*, *FOXP2* and the mature neuron marker *MAP2* were elevated in the patient-derived cells on day 35 (Fig. 3F). On day 60, in all groups most of the neurons were MAP2^+^. Compared to the Normal and isogenic controls, the patient-derived neurons contained decreased proportions of SATB2^+^ (Fig. 3G, H) and CUX1^+^ upper-layer neurons (Fig. 3G, I). qRT-PCR analysis further confirmed reduction in upper-layer neurons on day 60 in the patient-derived cells (Fig. 3J). Consistently, while the percentage of CTIP2^+^ deep-layer neurons was dramatically increased in the day-60 patient-derived organoids compared to that in the isogenic controls (Fig. 2I), the fraction of SATB2^+^ upper-layer neurons was significantly decreased (Fig. 3K). These findings together indicate that *CUL4B* deficiency leads to imbalance between deep-layer neurons and upper-layer neurons.

### Lack of CUL4B enhances neuronal excitability in cerebral organoids

**Fig. 4:**
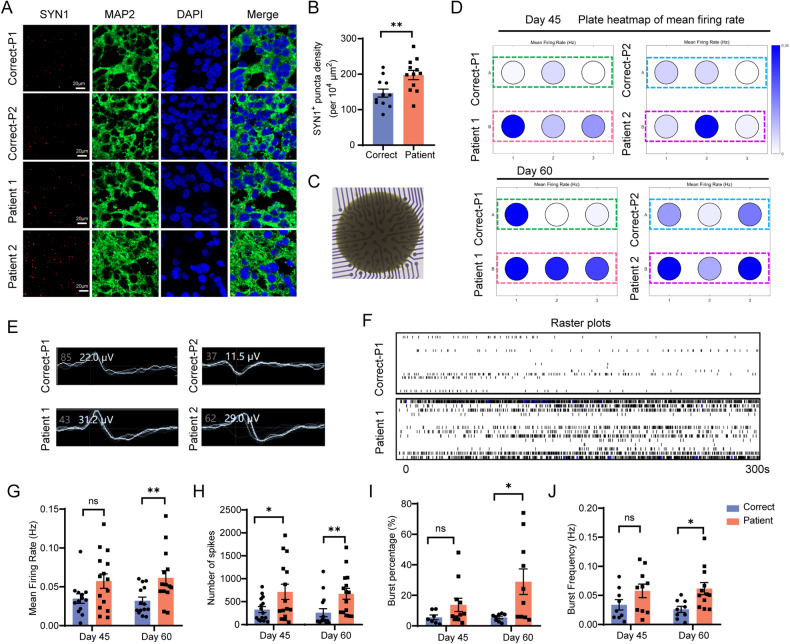
*CUL4B* deficiency enhances synapse formation and neuronal excitability. **A** Representative images of sections from the day-60 cerebral organoids stained with SYN1 and MAP2. Scale bar, 20 μm. **B** Quantification of SYN1^+^ puncta density in both the isogenic control (Correct) and the patient-derived (Patient) organoids on day 60. N = 12. **C** Representative bright-field image of a day-45 organoid plated on a 64-electrode array in an MEA well. **D** Plate maps of mean firing rates of both Correct and Patient organoids on day 45 and day 60. **E** Representative wave forms of spiking behavior from a single electrode for each Correct or Patient organoid on day 60. **F** Representative raster plots of Correct and Patient organoids (day 60) over 5-min continuous recording. Quantification of mean firing rate (**G**), number of spikes (**H**), burst percentage (**I**) and burst frequency (**J**) based on data obtained from Correct and Patient organoids on day 45 and day 60 over 10 min recording. A bundle of more than 10 spikes within 100 ms was defined as a burst. N = 8 MEA wells per genotype. Data are presented as the mean ± SEM. The statistical significance was determined using two-tailed unpaired t-test. *P < 0.05; **P < 0.01; ***P < 0.001. ns: no significance.

We next investigated whether *CUL4B* deficiency affects synapse formation and neuronal function in organoids. We quantified the density of synaptic puncta by staining the day-60 organoids with presynaptic vesicle protein synapsin 1 (SYN1) and dendrite marker MAP2. The density of SYN1^+^ puncta in dendrites was increased in the patient-derived organoids compared to that in the isogenic controls (Fig. 4A, B), suggesting accelerated functional maturation in organoids lacking CUL4B. We further accessed neuronal function in brain organoids using multi-electrode arrays (MEAs) (Fig. 4C). Extracellular spontaneous electrical activity was recorded from the day-45 and the day-60 organoids (Fig. 4D–F). We quantified the mean firing rate, the number of spikes, the burst percentage and the frequency of bursts. On day 60, all these parameters were significantly increased in the patient-derived organoids compared to the isogenic controls (Fig. 4G–J), indicative of elevated neuronal activity. Our results demonstrate that loss of CUL4B leads to neuronal hyperexcitability.

### The neurogenesis defects caused by *CUL4B* deficiency are due to inhibition of AKT and ERK activity

**Fig. 5:**
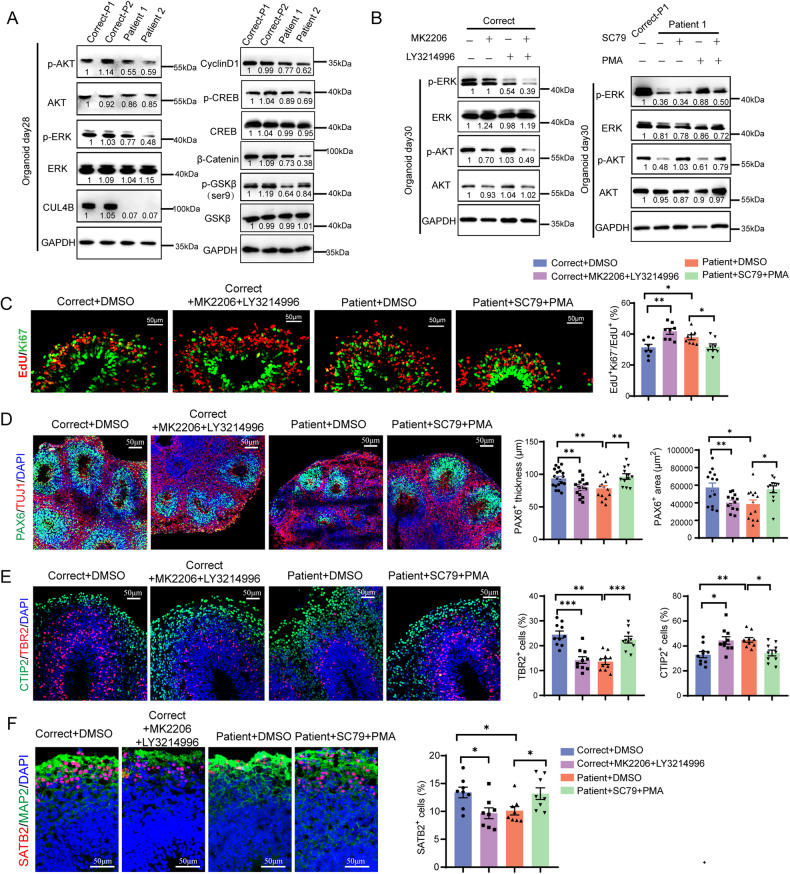
The neurogenesis defects in the patient-derived organoids are due to suppressed AKT and ERK. **A** Western blots showing the expression of the indicated proteins in organoids. **B** Western blots showing phosphorylation of ERK and AKT in the isogenic control (Correct) or the patient-derived (Patient) organoids with the indicated treatment. The number below each band is the normalized ratio between the intensity of the band and the intensity of GAPDH band from the same sample. **C** Ki67 staining (green) of the indicated organoids at 48 h after EdU labeling (red). Scale bar, 50 μm. N = 8. **D** PAX6 and TUJ1 staining in the indicated organoids on day 35. The bar graphs show the thickness (N = 12-18) and the area (N = 12-14) of the PAX6^+^ layers in organoids. **E** CTIP2 and TBR2 staining in day-50 organoids. Scale bar, 50 μm. The bar graphs show the proportion of TBR2^+^ cells (N = 10) and the proportion of CTIP2^+^ cells (N = 10). **F** SATB2 and MAP2 staining in day-60 organoids. Scale bar, 50 μm. The bar graph shows the proportion of SATB2^+^ cells. N = 8. Data are presented as the mean ± SEM. The statistical significance was determined using two-tailed unpaired t-test. *P < 0.05; **P < 0.01; ***P < 0.001. ns: no significance.

To elucidate the molecular mechanisms by which loss of CUL4B induces neurogenesis defects, we performed bulk RNA-sequencing on Correct-P1 and Patient 1 NPCs. Expression of 1696 genes (846 upregulated and 850 downregulated, fold change >2, FDR < 0.05) in NPCs was substantially altered by loss-of-function mutation of *CUL4B* (Supplementary Fig. S4A, B). Gene Ontology (GO) analysis revealed that genes upregulated in Patient 1 NPCs were enriched in GO terms related to neuron differentiation and neurogenesis, while the downregulated genes were enriched in GO terms related to epithelium and epidermis development and cell-cell junction assembly (Supplementary figure S4C), consistent with the premature neuronal differentiation phenotype in the patient-derived NPCs. KEGG analysis revealed that the genes downregulated in the patient-derived NPCs were enriched in PI3K-AKT signaling and MAPK signaling (Supplementary figure S4D). Previous studies have shown that inhibition of AKT or ERK activity results in premature neuronal differentiation [25, 26]. We evaluated the activities of AKT and ERK in 2D culture and cerebral organoids. We found phosphorylation of AKT and ERK were both reduced in the patient-derived NPC culture or organoids compared to the controls (Fig. 5A, Supplementary figure S4E). We also observed downregulated phosphorylation of CREB, a downstream effector of ERK, and decreased phosphorylated GSK3β and β-catenin, which are downstream of AKT, in the patient-derived cells and organoids (Fig. 5A, Supplementary figure S4E). These results together indicate inhibition of AKT and ERK in *CUL4B* mutants.

To determine whether the premature cell cycle exit and precocious neuronal differentiation caused by *CUL4B* deficiency are due to suppression of AKT and ERK activation, we first treated the control organoids with AKT inhibitor MK2206 and ERK inhibitor LY3214996. Western blot analysis revealed that MK2206 and LY3214996 suppressed the phosphorylation of AKT and ERK, respectively (Fig. 5B). Inhibition of AKT and ERK in the control organoids phenocopied the *CUL4B* deficient organoids, exhibiting increased cell cycle exit (Fig. 5C) and reduced thickness and area of PAX6^+^ layers (Fig. 5D). Inhibition of both AKT and ERK also decreased TBR2^+^ cells while increased CTIP2^+^ neurons in the control organoids (Fig. 5E). These results were consistent with previous reports on the requirement of AKT and ERK for normal cortical development [27]. We then determined whether activation of both AKT and ERK in the patient-derived organoids can rescue the defects in neurogenesis. Western blotting showed SC79 and PMA increased phosphorylation of AKT and ERK, respectively (Fig. 5B). Importantly, activation of both AKT and ERK in the patient-derived organoids prevented the premature cell cycle exit (Fig. 5C) and rescued the thickness and area of PAX6^+^ layers (Fig. 5D). Activation of both AKT and ERK in the patient-derived organoids also restored the percentage of TBR2^+^ and CTIP2^+^ cells to the control level (Fig. 5E). Furthermore, inhibition of both AKT and ERK in the isogenic controls reduced the percentage of SATB2^+^ cells on day 60. On the other hand, activation of both AKT and ERK in the patient-derived organoids rescued the impaired generation of upper-layer neurons (Fig. 5F). Similar results were also obtained on 2D culture (Supplemental figure S5). These data together suggest the neurogenesis defects caused by *CUL4B* deficiency are due to suppression of AKT and ERK.

### CUL4B regulates AKT and ERK activity through repression of *PPP2R2B* and *PPP2R2C* transcription

**Fig. 6:**
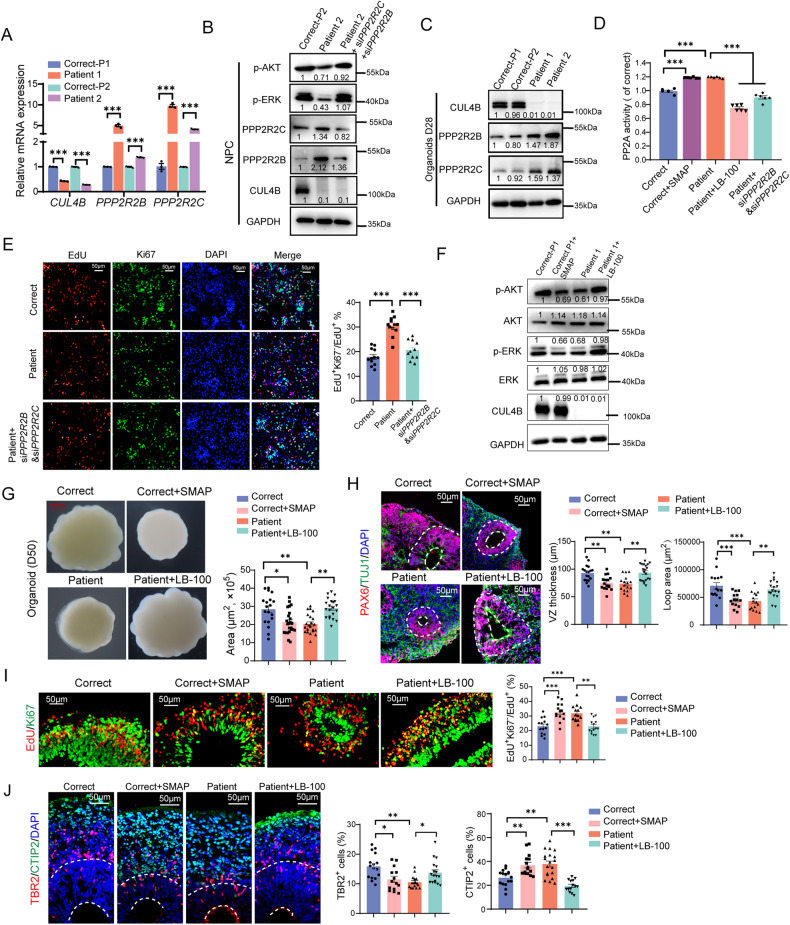
*CUL4B* deficiency impairs neurogenesis through activating PP2A. **A** qRT-PCR analysis of *PPP2R2B* and *PPP2R2C* expression in the isogenic control (Correct) and the patient-derived (Patient) NPCs. N = 3. **B** Western blots of p-AKT, AKT, p-ERK, ERK, PPP2R2B and PPP2R2C proteins in the indicated NPC cultures. **C** Western blots of PPP2R2B and PPP2R2C in day-28 organoids. **D** The PP2A activity in the indicated groups. N = 6. The values were normalized to the average in the Correct group. **E** Ki67 staining (green) of the indicated NPC cultures at 48 h after EdU labeling (red). Scale bar, 50 μm. N = 12. **F** Western blots showing the effect of LB-100 and SMAP treatment on phosphorylation of AKT and ERK in organoids. In (**B**, **C** and **F**), the number below each band is the normalized ratio between the intensity of the band and the intensity of GAPDH band from the same sample. **G** The effect of LB-100 and SMAP treatment on the size of day-50 cerebral organoids. Each data point represents the area of a single organoid. N = 20. **H** Quantification of the thickness and area of the PAX6^+^ layer in day-35 organoids. N = 14-20. **I** Ki67 staining (green) of the indicated organoids at 48 h after EdU labeling (red). Scale bar, 50 μm. N = 14. **J** Quantification of the percentage of CTIP2^+^ cells and TBR2^+^ cells in day-55 organoids. 14 to 20 neural tube-like regions in at least 7 organoids per group were counted. Data are presented as the mean ± SEM. The statistical significance was determined using one-way ANOVA with Tukey test. *P < 0.05; **P < 0.01; ***P < 0.001. ns: no significance.

Our next question is how *CUL4B* deficiency suppresses AKT and ERK activities. Both AKT and ERK can be dephosphorylated by protein phosphatase 2 A (PP2A) complex [28]. RNA sequencing revealed upregulation of *PPP2R2B* and *PPP2R2C*, which encode two isoforms of the regulatory subunit of PP2A, in the patient-derived NPCs (Supplementary figure S4B). We confirmed the increased expression of *PPP2R2B* and *PPP2R2C* by qRT-PCR in the patient-derived NPCs (Fig. 6A). The protein levels of PPP2R2B and PPP2R2C were also upregulated in both the patient-derived 2D NPC cultures and cerebral organoids (Fig. 6B, 6C). Upregulation of the regulatory subunit can cause increased PP2A complex activity [29]. Consistently, we detected upregulated PP2A activity in the patient-derived NPC cultures, which was rescued by interfering both *PPP2R2B* and *PPP2R2C* with siRNA (Fig. 6D). Moreover, knocking down *PPP2R2B* and *PPP2R2C* restored phosphorylation of AKT and ERK (Fig. 6B) and prevented the premature cell cycle exit in the patient-derived NPCs (Fig. 6E). We also evaluated the effect of a PP2A-specific inhibitor LB-100. Treatment with 1 μM LB-100 did not affect the viability of NPCs (Supplementary figure S6A), but suppressed the elevated PP2A activity in the patient-derived cells (Fig. 6D). Importantly, LB-100 treatment increased the phosphorylation of AKT and ERK in the patient-derived cells to the control level (Supplementary figure S6B), prevented the premature cell cycle exit (Supplementary figure S6C), and rescued the unbalanced generation of the upper-layer and the deep-layer neurons (Supplementary figure S6D, S6E). To further determine the role of PP2A in the neurogenesis defect caused by *CUL4B* deficiency, we applied LB-100 to the patient-derived organoids. Similar to what we observed in the NPC cultures, inhibition of PP2A in the patient-derived organoids restored phosphorylation of AKT and ERK (Fig. 6F) and rescued the defects in the organoid size (Fig. 6G), the VZ area (Fig. 6H), cell cycle exit (Fig. 6I) and neurogenesis (Fig. 6J). On the other hand, treating the control organoids with the PP2A activator SMAP resulted in decreased phosphorylation of AKT and ERK (Fig. 6D, F), reduced organoid size (Fig. 6G) and thinner PAX6^+^ layer (Fig. 6H) as well as premature cell cycle exit (Fig. 6I) and precocious neuronal differentiation (Fig. 6J), phenocopying the patient-derived organoids. These results together indicate that *CUL4B* deficiency impairs neurogenesis by activating PP2A complex.

**Fig. 7:**
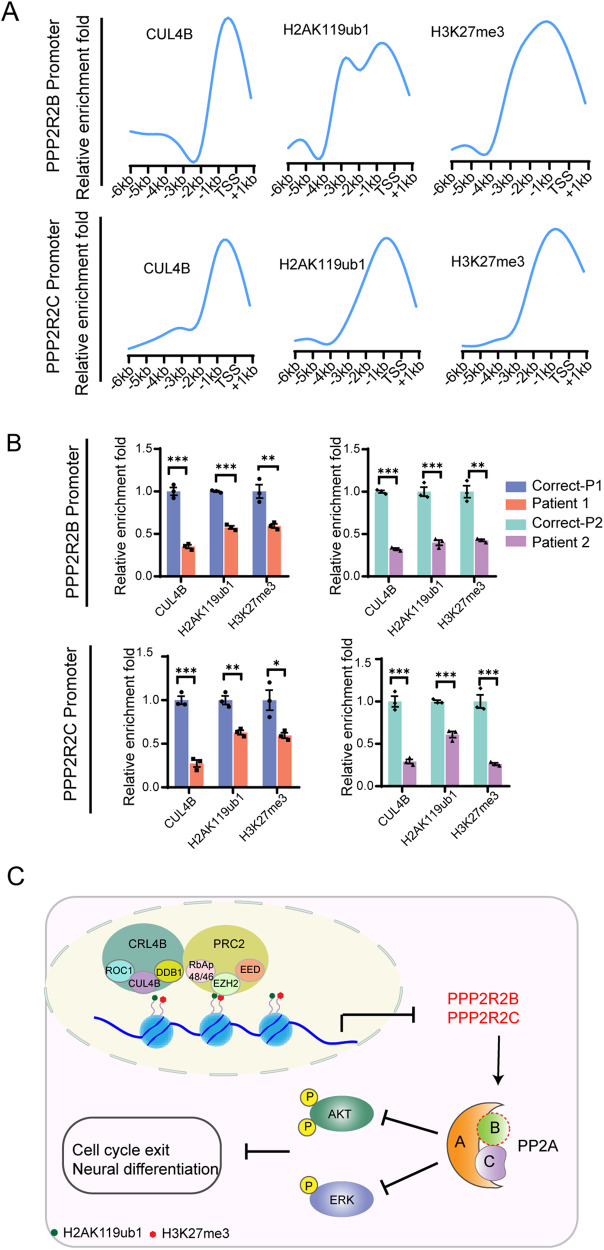
The CRL4B complex directly represses transcription of *PPP2R2B* and *PPP2R2C*. **A** qChIP results showing the enrichment patterns of CUL4B, H2AK119ub1 and H3K27me3 in the promoter of *PPP2R2B* and *PPP2R2C* in NPCs. Data are represented as the fold-change over control (IgG). **B** qChIP analysis of enrichment of CUL4B, H2AK119ub1 and H3K27me3 in *PPP2R2B* promoter and *PPP2R2C* promoter in Correct and Patient NPCs. N = 3. Data are presented as the mean ± SEM. The statistical significance was determined using two-tailed unpaired t-test. *: *P* < 0.05; **: *P* < 0.01; ***: *P* < 0.001. ns: no significance. **C** Schematic showing the mechanism underlying CUL4B regulation of neurogenesis. In wild type NPCs, CRL4B complex cooperates with PRC2 complex to repress transcription of *PPP2R2B* and *PPP2R2C*. In NPCs with *CUL4B* deficiency, *PPP2R2B* and *PPP2R2C* are de-repressed, leading to upregulated PP2A complex. PP2A complex then inhibits AKT and ERK, resulting in premature cell cycle exit and precocious neuron differentiation.

How does CUL4B regulate the expression of *PPP2R2B* and *PPP2R2C*? CRL4B complex can function as a transcriptional corepressor through catalyzing monoubiquitination of H2AK119 and recruitment of PRC2 complex [12]. To investigate whether *PPP2R2B* and *PPP2R2C* are direct targets of CRL4B, we performed chromatin immunoprecipitation (ChIP). We detected enrichment of CUL4B in the promoters of *PPP2R2B* and *PPP2R2C* (Fig. 7A). We also found enrichment of H2AK119ub1 and H3K27me3, which are catalyzed by CRL4B complex and PRC2 complex, respectively, in the same region (Fig. 7A). The enrichment of H2AK119ub1 and H3K27me3 in *PPP2R2B* and *PPP2R2C* promoters were dramatically reduced in the patient-derived NPCs compared to that in the isogenic controls (Fig. 7B), suggesting that CRL4B cooperates with PRC2 complex to repress *PPP2R2B* and *PPP2R2C* transcription.

## Discussion

Mutation in *CUL4B* is one of the most common causes of XLID [30, 31]. In this study, using the patient-derived iPSCs, we developed 2D and 3D in vitro models of XLID caused by *CUL4B* c.1564 C > T mutation or *CUL4B* c.1007_1011del mutation. We demonstrated that loss-of-function *CUL4B* mutations led to impaired cortical neurogenesis, as shown by premature cell cycle exit, precocious and unbalanced neuronal differentiation and neuronal hyperexcitability. Mechanistic studies revealed that CRL4B complex epigenetically repressed *PPP2R2B* and *PPP2R2C* expression by catalyzing H2AK119 monoubiquitination. Loss of CUL4B increased PP2A activity through upregulating PPP2R2B and PPP2R2C, which inhibited ERK and AKT activities in NPCs to drive cell cycle exit and neuronal differentiation (Fig. 7C). Our work provides the first evidence that CUL4B regulates neurogenesis during brain development.

In recent years, human brain organoids have been successfully used to study the pathogenesis of neurodevelopmental disorders like Down syndrome, fragile X syndrome, Miller-Dieker syndrome [32–35]. In the present study, we found impaired growth of the patient-derived *CUL4B*-deficient cerebral organoids, which was consistent with microcephaly presented in our patients [3]. Recently, Stier et al. generated a null *CUL4B* mutation in H9 embryonic stem cells and showed that the mutation resulted in severe loss of ventricular structures in brain organoids. The mutant organoids barely had ventricular structures by day 35 [36]. However, in our study, we still observed many ventricular structures in the day-60 patient-derived organoids although they were smaller than those in the isogenic controls. The difference between the work from Stier et al. and ours may be due to the different types of cell lines used (H9 embryonic stem cells vs. patient-derived iPSCs) and the nature of the mutations.

CUL4B has been reported to promote proliferation in diverse cell types [37–39]. Previously, Liu et al. reported that reduced CUL4B expression resulted in G2/M arrest in primary rat NPCs and human NT2 cells [40]. In this study, we found proliferation in the patient-derived NPCs was similar to that in the control NPCs on day 16 of 2D neuronal differentiation, when NPCs were expanding, but decreased on day 25, when neuronal differentiation of NPCs was initiated, suggesting that CUL4B may be essential for preventing precocious differentiation rather than promoting proliferation of NPCs. EdU incorporation and tracing analysis on 2D culture and cerebral organoids further demonstrated that the fate choices of *CUL4B*-deficient NPCs were biased toward neuronal differentiation as opposed to the continued self-renewal observed in control cells. The premature cell cycle exit and precocious neuronal differentiation can gradually deplete the NPC pool, which may explain the impaired growth of the patient-derived organoids.

In this study, we demonstrated that the neuronal cultures and cerebral organoids derived from the patients with *CUL4B* mutations contained more deep-layer neurons and fewer upper-layer neurons compared to their isogenic controls. The development of human cerebral cortex from NPCs is a dynamic, highly regulated process in which different classes of neurons are produced in a fixed temporal order [41, 42]. The precocious transition from self-renewal to neuron differentiation in NPCs often causes imbalance between upper-layer neurons and deep-layer neurons [43]. In addition, we observed dramatically reduced TBR2^+^ cells, which are outer radial glia cells (oRG/bRG) and transit-amplifying intermediate progenitor (IP) cells, in the patient-derived organoids. These progenitor cells have been proposed to contribute to the majority of upper-layer neurogenesis [44].

To investigate the molecular mechanism underlying the impaired neurogenesis caused by *CUL4B* mutations, we performed RNA sequencing and identified AKT and ERK as downstream mediators of CUL4B regulation of neurogenesis. Dysregulated ERK and AKT signaling have been linked to neurodevelopmental defects [27]. Previous study has shown that decreased activities of AKT and ERK lead to premature neural differentiation in cardiofaciocutaneous syndrome [45]. In Rett syndrome, inhibition of ERK and AKT signaling by MeCP2-regulated miRNA results in abnormal human neurogenesis [46]. ERK signaling is required for maintenance of neural progenitor population. Loss of ERK2 causes elongation of the cell cycle (G1 phase specifically) due to decreased levels of Cyclin D1 [26, 47]. We found that exposure of the control NPCs or organoids to AKT and ERK inhibitors mimicked the premature cell cycle exit and unbalanced neuron differentiation observed in *CUL4B* mutants. More importantly, activation of AKT and ERK in the *CUL4B* mutants was able to rescue the neurogenesis defects, supporting that CUL4B regulates neurogenesis via modulation of AKT and ERK signaling.

Epigenetic regulation plays important roles in neurodevelopment. It has been reported that CRL4B complex mediates ubiquitination and degradation of WDR5, a core subunit of histone H3 lysine 4 (H3K4) methyltransferase complex, to regulate neuronal gene expression [48]. Our previous studies have shown that CRL4B complex catalyzes H2AK119 monoubiquitylation and recruits PRC2 complex and/or SIN3A-HDAC complex to repress gene transcription [12, 38]. In this study, we identified CRL4B complex as an epigenetic repressor of the transcription of *PPP2R2B* and *PPP2R2C* in NPCs. *PPP2R2B* and *PPP2R2C* encode two isoforms of the regulatory B subunit of PP2A and regulates the PP2A activity [49]. Knocking down both *PPP2R2B* and *PPP2R2C* or inhibition of PP2A activity in *CUL4B* mutant NPCs largely restored the phosphorylation of AKT and ERK, and rescued neurogenesis defects in *CUL4B*-deficient organoids, supporting that CUL4B regulates AKT and ERK signaling via suppression of PP2A. Repression of *PPP2R2B* and other components of PP2A complex by CRL4B complex have been reported in our previous work on hepatocellular carcinoma [50] and myeloid derived suppressive cells (MDSCs) [9], suggesting PP2A as a common downstream target of CRL4B in different biological processes. Intriguingly, both PPP2R2B and PPP2R2C have been associated to neurological disorders. Disruption of *PPP2R2C* gene may be the cause for intellectual disability in a family with reciprocal translocation (4;6)(p16.1;q22) [51]. Expansion of CAG repeats in *PPP2R2B* gene has been associated with autosomal dominant spinocerebellar ataxia 12, characterized by diffuse cerebral and cerebellar atrophy [52]. These studies support the critical role of CUL4B-PP2A axis in neurodevelopment.

In conclusion, with forebrain NPCs and cerebral organoids generated from the patient-derived iPSCs, we demonstrate that CUL4B prevents premature cell cycle exit and precocious neuron differentiation in NPCs through suppression of PP2A and the subsequent activation of AKT and ERK. Our work provides mechanistic insights into the pathogenesis of the neurological disorders in XLID patients with *CUL4B* mutations.

## Materials and methods

### Generation of human iPSCs from PBMCs

Normal iPSC lines were generated from human peripheral blood mononuclear cells (PBMCs) obtained from two male healthy donors (Supplementary Table S1). 2 μg of episomal expression plasmid mixture encoding reprogramming factors OCT4, SOX2, KLF4, MYC and Lin28 were electroporated into 1 × 10^6^ PBMCs using 2b Nucleofector (Lonza, Cologne, Germany) with CD34 Cell Nucleofector Kit (Lonza, Cat#VPA-1003) according to the manufacturer’s instructions. Colonies were picked up 25 days after electroporation, and the colonies similar to hESCs were selected for further cultivation and evaluation.

### Culture of human iPSCs and gene-editing using CRISPR-Cas9

iPSCs were maintained on Matrigel (Corning, Cat#354277)-coated plates with mTesR1 medium (StemCell Technologies, Cat# 85850), and passaged using Gentle Cell Dissociation Reagent (StemCell Technologies, Cat# 100-1077). All iPSCs used in this study were below passage 40 and confirmed mycoplasma free.

For genome editing, guide-RNAs (gRNAs) targeting *CUL4B* c.1564 C > T or c.1007-1011del site were designed using CRISPOR (http://crispor.tefor.net/) based on hg38 reference. gRNA oligonucleotides were then cloned into pSpCas9 (BB)-2A-GFP (PX458) plasmid (Addgene plasmid # 48138). We designed single-strand oligo-deoxynucleotide (ssODN) templates carrying correct sequence and two silent mutations within sgRNAs binding region in close proximity to the protospacer adjacent motif (PAM) site to prevent the templates from recurrent cleavage in edited cells. Plasmids and donor templates were introduced into the patient-derived iPSCs through nucleofection using Amaxa nucleofector II (Lonza Nucleofector) according to the manufacturer’s instructions. GFP-positive cells were collected using the cell sorter (Beckman MoFlo, Beckman Coulter) within 72 h post electroporation. Single-cell-derived colonies were manually picked and further expanded. Genomic DNAs were isolated from each iPSC colony and genotyped using Sanger sequencing on PCR amplicons. For analysis of off-target effects of CRISPR-Cas9 genome editing, we used the Cas-offinder (https://www.rgenome.net/cas-offinder) for rigorous prediction of off-target sites, and the top 8 possible off-target sites in genome were all checked by sanger sequencing (Supplementary Table S2). Primers, sgRNA, and HDR templates sequences are listed in Supplementary Table S3.

### Differentiation of iPSCs into forebrain-specific NPCs and cortical neurons

We obtained NPCs and neurons using a previously published protocol with minor modifications [22]. Briefly, iPSCs were seeded onto Matrigel-coated plates in mTeSR1 medium at a density of 10^6^ cells/ml. In the next day, the medium was replaced with N2B27 medium containing 50% DMEM/F12 (Invitrogen, Cat#11330-032), 0.5× N2 supplement (Invitrogen, Cat#17502048), 50% Neurobasal (Invitrogen, Cat#21103049), 0.5× B27 (Invitrogen, Cat#17504044), 1% GlutaMax (Invitrogen, Cat#35050-038), 1% MEM-NEAA (Invitrogen, Cat#11140-050), 5 μg/ml insulin (Sigma, Cat#19278), and 1 μg/ml heparin (Sigma, Cat#H3149) plus 5 μM SB431542 (Sigma, Cat#S4317) and 5 μM Dorsomorphin (Sigma, Cat#P5499). After 8 days induction, cells were passaged at 1:2 ratio in N2B27 medium. Upon appearance of rosettes, 20 ng/ml of FGF2 (PeproTech, Cat#100-18B) was added for 4 days. After 16 days, neural rosettes were mechanically dissociated and plated on matrigel-coated dishes in N2B27 medium. For neuron random differentiation, NPCs were digested into single cells with Accutase and seeded on poly-l-ornithine/laminin (Sigma)-coated dishes in neuronal medium containing N2B27 medium plus 20 ng/ml GDNF (Peprotech, Cat#450-10), 20 ng/ml BDNF (Peprotech, Cat#450-02), and 1 mM cAMP (Sigma, Cat#A9501). Neuronal medium was changed every 2 days.

### Organoid culture

We followed a previously described protocol with minor modifications [23, 53]. In brief, iPSCs were dissociated to single cells with Accutase, and seeded in 96-well plates at 9000 cells per well with embryoid body (EB) medium containing DMEM/F12, 20% Knockout Serum Replacement (KSR) (Invitrogen, Cat#10828-028), 1% GlutaMax, 1% MEM-NEAA, 0.1 mM 2-mercaptoethanol (Invitrogen, Cat#21985023), 20 μM ROCK inhibitor Y-27632 (StemCell Technologies, Cat#72304). After 5 days, healthy EBs were transferred to 24-well ultralow attachment plates (Corning) in neuron induction medium consisting of DMEM/F12, 1% N2, 1% MEM-NEAA and 1 μg/ml heparin. After about seven days, the EBs that developed translucent edges were embedded in Matrigel and transferred to ultra-low-attachment 6-well plates in differentiation medium, which contained 50% DMEM/F12, 50% neurobasal, 0.5% MEM-NEAA, 0.5% GlutaMax, 0.5% N2, 1% B27, 2.5 μM insulin, and 100 μM β-mercaptoethanol. B27 without vitamin A (Invitrogen, Cat#12587010) were used in differentiation medium for four days, then replaced with B27 containing vitamin A. Organoids were further cultured under orbital agitation (80 rpm) with medium change every 4 days. In order to yield high-quality organoids, 10 μM SB431542 and 1 μM Dorsomorphin were added during the neural induction phase. To evaluate the effect of altering activities of AKT, ERK or PP2A, the organoids were treated with 1 μM MK2206 (Selleck, Cat# S1078) plus 10 μM LY3214996 (MCE, Cat#HY-101494), or 10 μg/ml SCH79 (MCE, Cat#HY-18749) plus 1 μM PMA (MCE, Cat# HY-18739) from day 25 to day 35, 5 μM SMAP (MCE, Cat#HY-112929) or 1 μM LB-100 (Selleck, Cat#S7537) from day 25 up to day 55. To label proliferative progenitors, organoids were incubated with 10 μM EdU (Beyotime, Cat# C0078S) for 2 h, followed by extended culture in organoid differentiation medium for 2 days.

### Immunofluorescent staining of cerebral organoids and 2D cells

Cerebral organoids were fixed in 4% paraformaldehyde at 4 °C overnight, cryoprotected in 30% sucrose solution, embedded in optimum cutting temperature (OCT) compound (Tissue Tek), then cryosectioned at 14 μm thickness. 2D cells on coverslips were fixed in 4% paraformaldehyde for 15 min. The sections or cell coverslips were washed three times with PBS, blocked and permeabilized for 1 h at RT with 5% BSA (Sigma) and 0.5% Triton X-100 (Sigma), then incubated with primary antibodies overnight at 4 °C, followed by washing with PBS and subsequent 1 h incubation with secondary antibodies (1:250). Cells were counterstained with DAPI (Sigma). Fluorescence images were captured using the confocal microscope (Zeiss LSM 980, Carl Zeiss, Germany) and analyzed using ImageJ software (NIH, Rockville, MD, USA). All the used antibodies are listed in Supplementary Table S4.

### RNA sequencing

RNA was extracted from NPCs (Day 17) of Patient 1 and Correct-P1 using Trizol reagent (Thermo Fisher Scientific, Cat#15596-026) according to the manufacturer’s protocol. RNA quality was assessed on an Agilent 2100 Bioanalyzer (Agilent Technologies, Palo Alto, CA, USA) and was reversely transcribed into cDNA using NEBNext Ultra RNA Library Prep Kit for Illumina (NEB#7530, New England Biolabs, Ipswich, MA, USA). The resulting cDNA library was sequenced using Illumina Novaseq6000 by Gene Denovo Biotechnology Co. (Guangzhou, China). RNA-seq data were mapped to Gencode GRCh38.p12 Release 29 human reference genome using STAR aligner v2.6.0c. Differential expression analyses were performed using edgeR. The raw RNA-seq data are accessible at PRJNA945348.

### Multielectrode array analysis (MEA)

Organoids were placed into 6-well MEA plates (Axion Biosystems, Atlanta, GA, USA, Cat# M384-tMEA-6W) coated with poly-l-ornithine/laminin (Sigma) and with 64 platinum microelectrodes arrays per well. Recordings were collected with the Maestro MEA system (Axion Biosystems) at 37 °C and 5% CO_2_, for 15-20 min using the AxIS Software (Axion Biosystems). Data analysis was performed using Axion Biosystems’s Neural Metric Tool and AxIS Metric Plotting Tool. The spike detecting threshold was set to 5.5 standard deviations, and the electrodes with at least five spikes per minute were considered as active.

### Chromatin immunoprecipitation (ChIP) analysis

ChIP was performed as described previously [38]. Briefly, 1 ×10^7^ cells were crosslinked with 1% formaldehyde, sonicated, precleared and incubated with 5-10 μg of antibody per reaction overnight. The complexes were then rinsed with low-salt and high-salt buffers. After extraction and purification of DNA, qPCR was performed to analyze the enrichment of DNA template. Primers specific for the interested genomic regions are listed in Supplementary Table S3. The antibodies used for ChIP are listed in Supplementary Table S4.

### Cell viability assay

NPCs were seeded into 96-well plates (5 × 10^3^ cells/well) in triplicates and cultured in Neural Progenitor Medium with LB-100 at 1, 2, 3, 4, 5, 6, 7 or 8 μM for 24 h. 10 μl of CCK-8 solution (Beyotime, Cat#C0038) was added to each well. The cell viability was determined 1 h later by measuring the absorbance at 450 nm.

### Phosphatase activity assay

NPCs were washed with pre-chilled PBS and collected in RIPA lysis buffer. Then, the cells were centrifuged at 2000 g for 10 min at 4 °C. The supernatant was collected, and the protein concentration was measured. PP2A activity was determined using the Serine/Threonine Phosphatase Assay System (Promega, Cat# V2460) according to the manufacturer’s manual.

### Quantitative Real Time (qRT)-PCR

Total RNA was extracted using Trizol reagent according to the manufacturer’s recommendation. RNA quality and concentration were assessed using Nanodrop. RNA was reverse transcribed into cDNA using RT SuperMix (Vazyme, Cat# R323-01). Primers were designed with Primer3 software (version 0.4.0) (Supplementary Table S3). qPCR was performed using ChamQ SYBR qPCR Master Mix (Vazyme, Cat# Q411-03) on Roche 480 system. mRNA expression levels were calculated based on the 2^−ΔΔCT^ method relative to GAPDH levels. qPCR data were presented as the ratio relative to the average in the control groups.

### RNA interference

The NPCs were cultured in Neural Progenitor Medium (StemCell Technologies, Cat# 05833). Cells were transfected with small interfering RNAs (siRNAs) using the Lipofectamine RNAiMAX (Invitrogen) following the manufacturer’s instructions. The siRNAs against *PPP2R2B* and *PPP2R2C* were purchased from Shanghai Gene-Pharma Co., Ltd (Shanghai, China). The targeted sequences are listed in Supplementary Table S3.

### Western blot

Cells or organoids were lysed in RIPA buffer (Beyotime, Cat#P0013B) in the presence of protease and phosphatase inhibitors. Lysed samples were centrifuged for 20 min at 12,000 rpm at 4 °C. Protein concentration was determined using BCA assay kit (Thermo Fisher, Cat# 23225). 20 μg protein was separated in 10% SDS-PAGE gels, and subsequently transferred to PVDF membranes (Millipore). Membranes were blocked with 5% BSA for 1 h at RT and probed with primary antibodies overnight at 4 °C. Membranes were incubated with HRP-conjugated anti-rabbit antibody (1:5000) or anti-mouse antibody (1:5000) for 1 h at RT after washing with TBST. Protein bands were visualized using Chemiluminescent Substrates (Spark Jade, Cat#ED0016) and Tanon 5200 Multi Chemiluminescence imager (Tanon Science & Technology Co., Shanghai, China). The used antibodies are listed in Supplementary Table S4.

### Statistics

Data are presented as the mean ± standard error of the mean (SEM). Statistical analyses were performed using GraphPad Prism 9 software. Statistical significance was determined using the two-tailed unpaired t-test for two-sample comparison or one-way ANOVA for comparison between three or more samples. The Tukey test was used to derive adjusted *P*-value for multiple comparisons. *P* < 0.05 was considered as statistically significant. The assumption of equal variance was validated by F-test. The sample sizes were chosen empirically based on the observed effects and previous reports. The sample size for each experiment is listed in the figure legends. When collecting and analyzing data of RT-qPCR and immunostaining, the investigators were blinded to the group allocation. All the experiments were repeated at least three times. All the repeats were successful, and the representatives were shown in the figures.

### Supplementary information

Supplementary information

Original Data File

reporting checklist

## Data Availability

The raw data for RNAseq can be assessed at PRJNA945348. All the other raw data supporting the findings of this study are available from the corresponding authors upon request.
