# Supplementary material for: CUL4B mutations impair human cortical neurogenesis through PP2A-dependent inhibition of AKT and ERK
Source: Cell Death Dis. 2024 Feb 8;15(2):121. doi: 10.1038/s41419-024-06501-3 (PMC10853546; doi:10.1038/s41419-024-06501-3)
Supplement: Supplementary file 1 — Supplementary information [file 41419_2024_6501_MOESM1_ESM.pdf]

***CUL4B* mutations impair human cortical neurogenesis through PP2A-dependent inhibition of AKT and ERK**

Ma et al.

This file contains the following contents.

Supplementary Figure S1-S6

Supplementary Table S1-S4

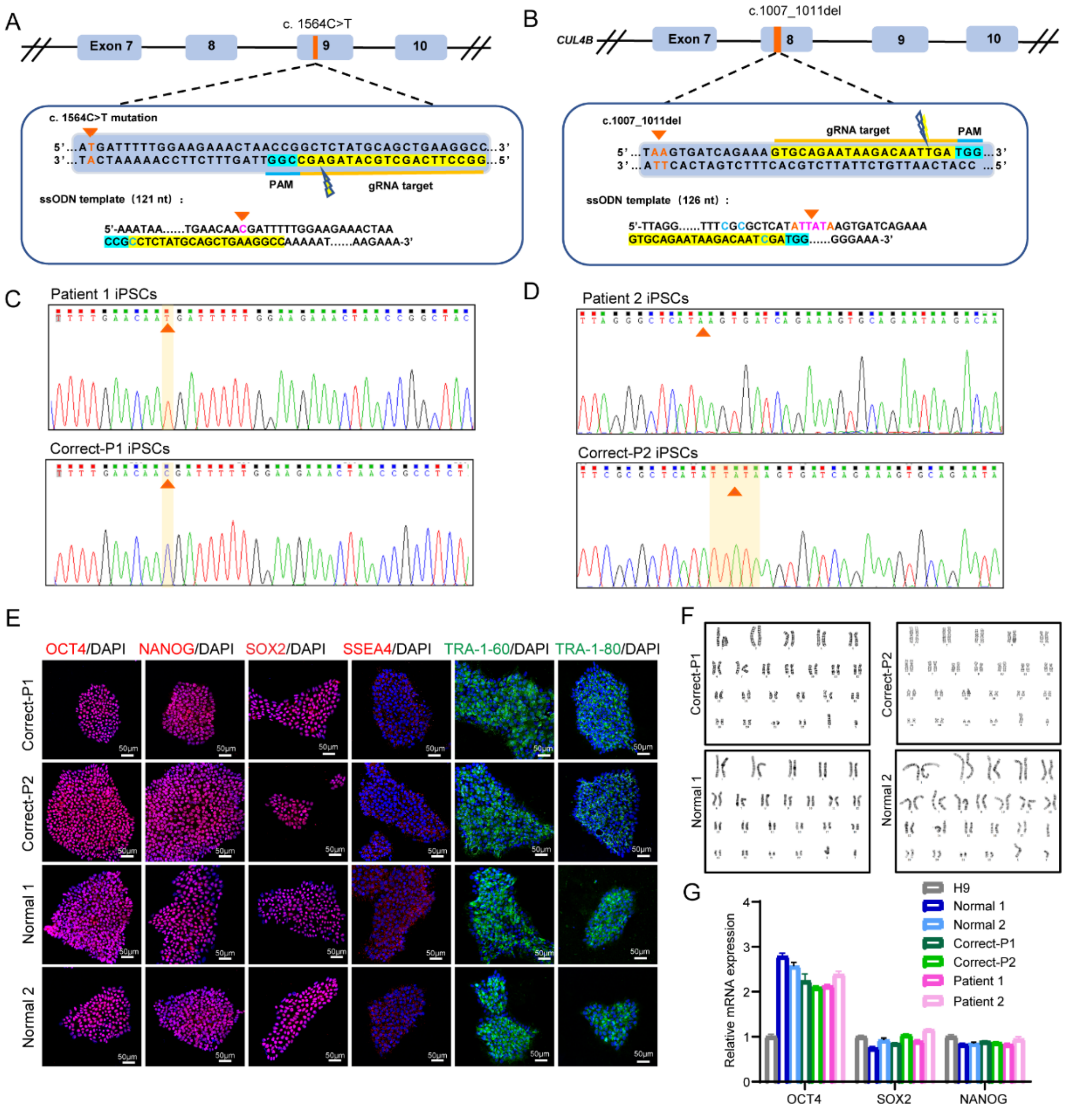

Supplementary Figure S1. *CUL4B* deficiency does not affect the pluoripotency of human iPSCs.

A & B) CRISPR/Cas9 knock-in strategy for *CUL4B* mutation c.1564C>T (A) and c.1007_1011del (B). Yellow highlights the guide RNA sequence. C) Sequencing results around *CUL4B* c.1564C>T mutation site before and after gene correction. D) Sequencing results around *CUL4B* c.1007_1011del mutation site before and after gene correction. Orange arrowhead in (C) & (D): corrected mutations. E) Immunostainings of pluripotency markers OCT4, NANOG, SOX2, SSEA4, TRA-1-60 and TRA-1-80 in

the indicated iPSCs. F) Karyotype analysis of the indicated iPSC lines. G) qRT-PCR of the pluripotency markers in the indicated iPSC lines. Human embryonic stem cell line H9 was used as positive control. N=4, data are presented as the mean ± SEM.

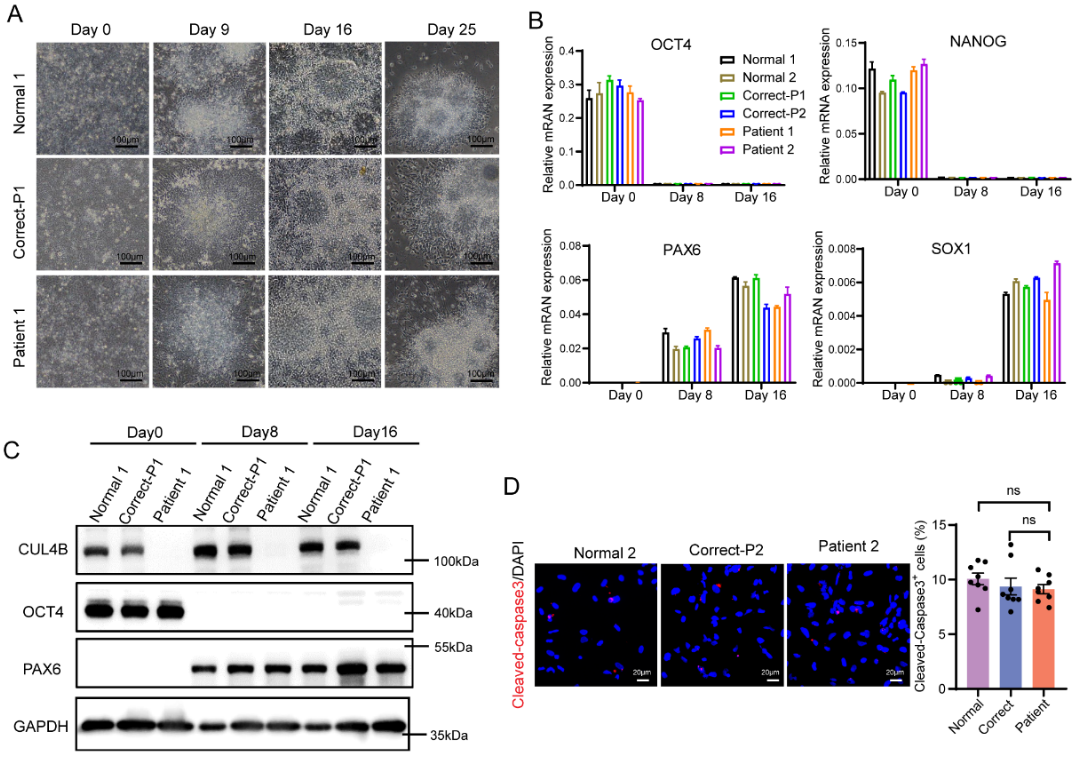

Supplementary Figure S2. Neither differentiation of iPSCs to NPCs nor cell death of NPCs is affected by *CUL4B* deficiency.

A) Images of cells in the process of neural differentiation of iPSCs. Scale bar, 100 µm. B) qRT-PCR analysis of the expression of the pluripotent genes OCT4, NANOG and the NPC markers PAX6, SOX1 on day 0, day 8 and day 16 of neural differentiation. N=3. C) Western blots showing protein levels of OCT4, PAX6 and CUL4B in the indicated cells on day 0, day 8 and day 16 of neural differentiation. D) Cleaved caspase-3 staining in NPCs. Scale bar, 20 µm. N=8. The statistical significance was determined using one-way ANOVA with Tukey test. ns: no significance.

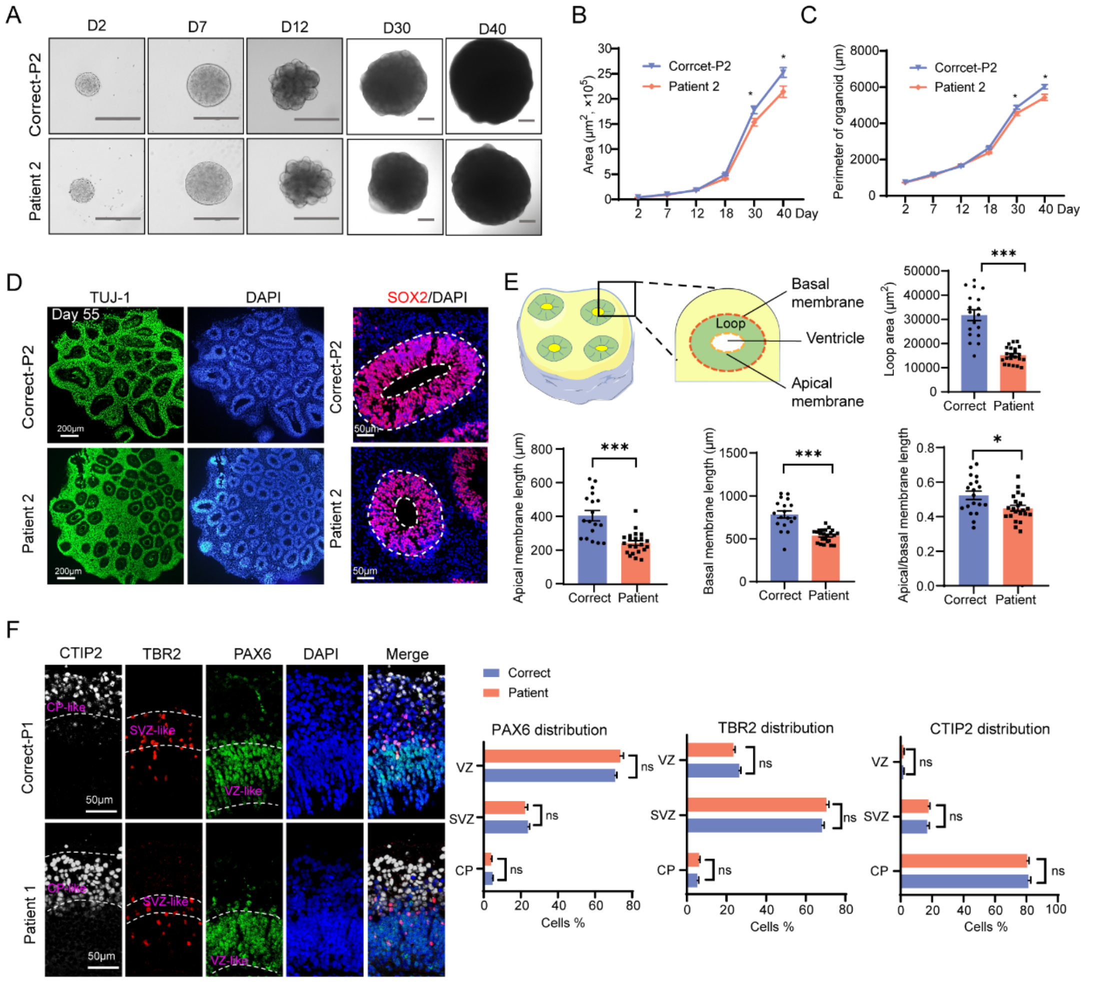

Supplementary Figure S3. ***CUL4B*** **mutations impair the growth and structure of cerebral organoids.**

A) Bright-field images of Correct-P2 and Patient 2 brain organoids at the embryoid body stage (Day 1 and Day 7) and differentiation stage (Day 12, Day 18, Day 30 and Day 40). Scale bar, 400 μm. B-C) Quantifications of the area (B) and the parameter (C) of organoids at different developmental time points. More than 15 organoids from three independent experiments were analyzed for each line. D) Representative images of TUJ1 and SOX2 staining of day-55 organoids. Scale bars on the left, 200 μm. Scale bars on the right, 50 μm. E) Schematic of the structure of neuroepithelial loops in organoids and the quantifications of the indicated parameters. In the schematic, loop

tissues are shown in green. The white region circled by the orange dotted line is ventricle. n=15 to 22 regions in at least 5 organoids per cell line were measured. F) Representative images of Correct-P1 and Patient 1 cortical organoids immunostained with CTIP2, TBR2 and PAX6 antibodies. Scale bar, 50 μm. The percentages of $PAX6^+$, $TBR2^+$ cells and $CTIP2^+$ cells located in VZ, SVZ and CP were analyzed. N=12. Data are presented as the mean ± SEM. The statistical significance was determined using two-tailed unpaired t-test. *: $P < 0.05$; **: $P < 0.01$; ***: $P < 0.001$. ns: no significance.

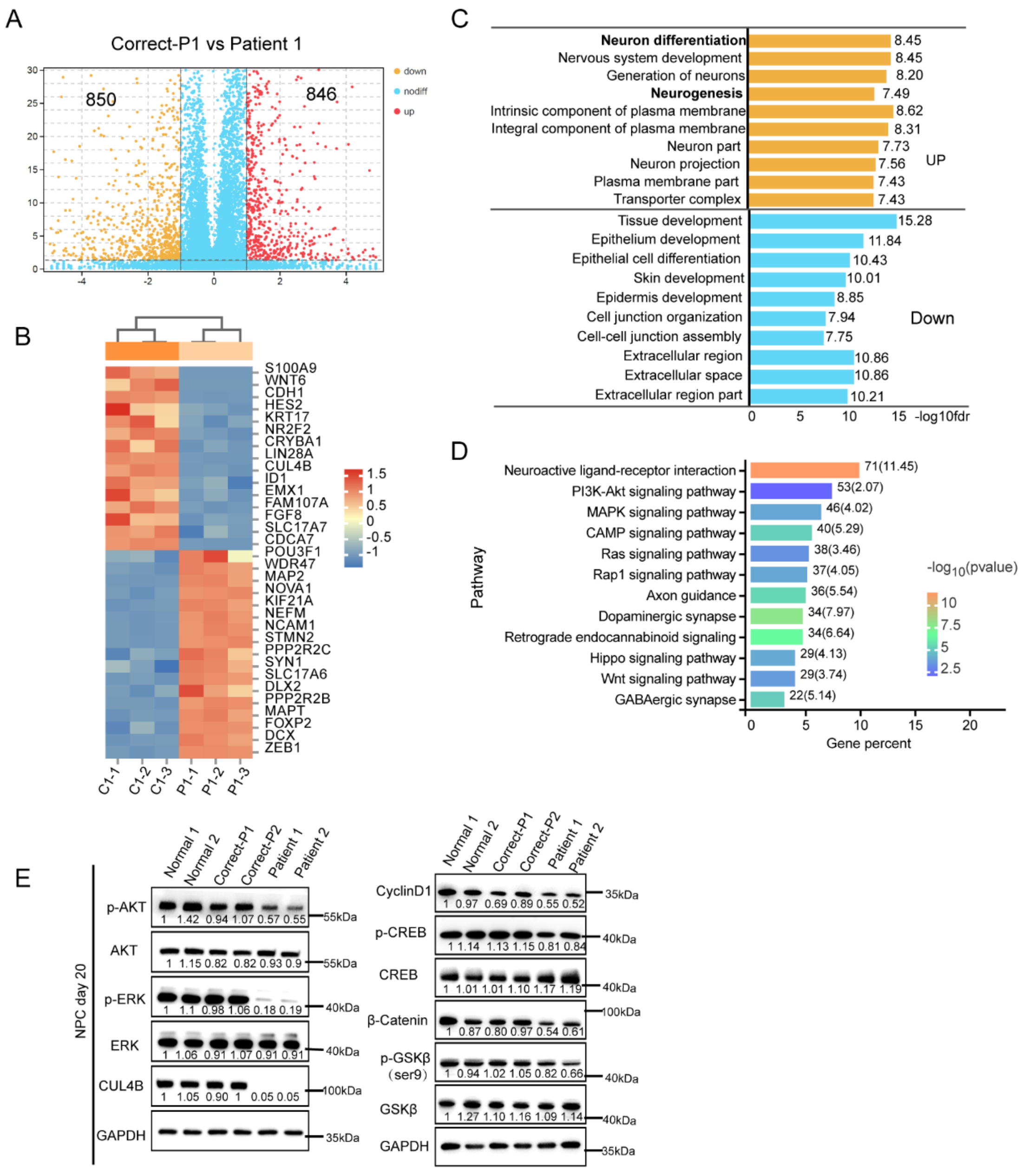

Supplementary Figure S4. *CUL4B* deficiency leads to activation of AKT and ERK.

A) Volcano plot showing the number of genes differentially expressed in Correct-P1 and Patient 1 NPCs. Yellow dots are downregulated genes and red dots are upregulated genes ($p < 0.05$, fold change >2). B) Heatmap of the representative differentially expressed genes (DEGs) involved in neurodevelopment. P1: Patient 1. C1: Correct-P1. C) Top 10 GO terms for biological process enriched with genes upregulated or downregulated in Patient 1 NPCs. D) KEGG pathway enrichment analysis for genes

downregulated in Patient 1 NPCs. E) Western blots showing the expression of the indicated proteins in NPCs. The number below each band is the normalized ratio between the intensity of the band and the intensity of GAPDH band from the same sample.

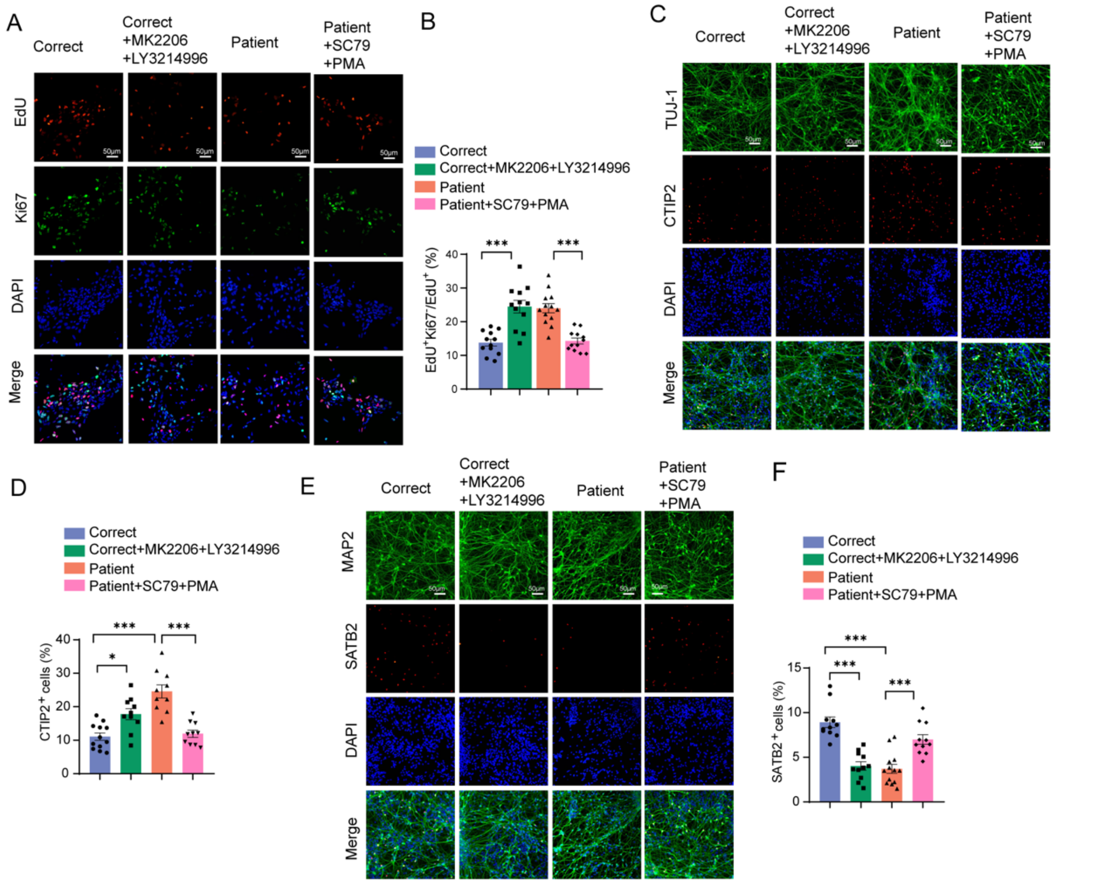

Supplementary Figure S5. Neurogenesis defects in the patient-derived NPCs are due to inhibition of AKT and ERK.

A) Ki67 staining (green) of the indicated NPCs at 48 h after EdU labeling (red). Scale bar, 50 µm. B) Quantification of the percentage of EdU^+ $Ki67^-$ in EdU^+ cells. N=12. C) Representative images of TUJ-1 and CTIP2 staining in day-40 neural culture. D) Quantification of the percentage of $CTIP2^+$ cells in different groups. N=10-13. E) Representative images of MAP2 and SATB2 staining in day-60 neural culture. Scale bar, 50 µm. F) Quantification of the percentage of $SATB2^+$ cells in different groups. N=10-13. Data are presented as the mean ± SEM. The statistical significance was determined using one-way ANOVA with Tukey test. *: $P< 0.05$; **: $P< 0.01$; ***: $P< 0.001$. ns: no significance.

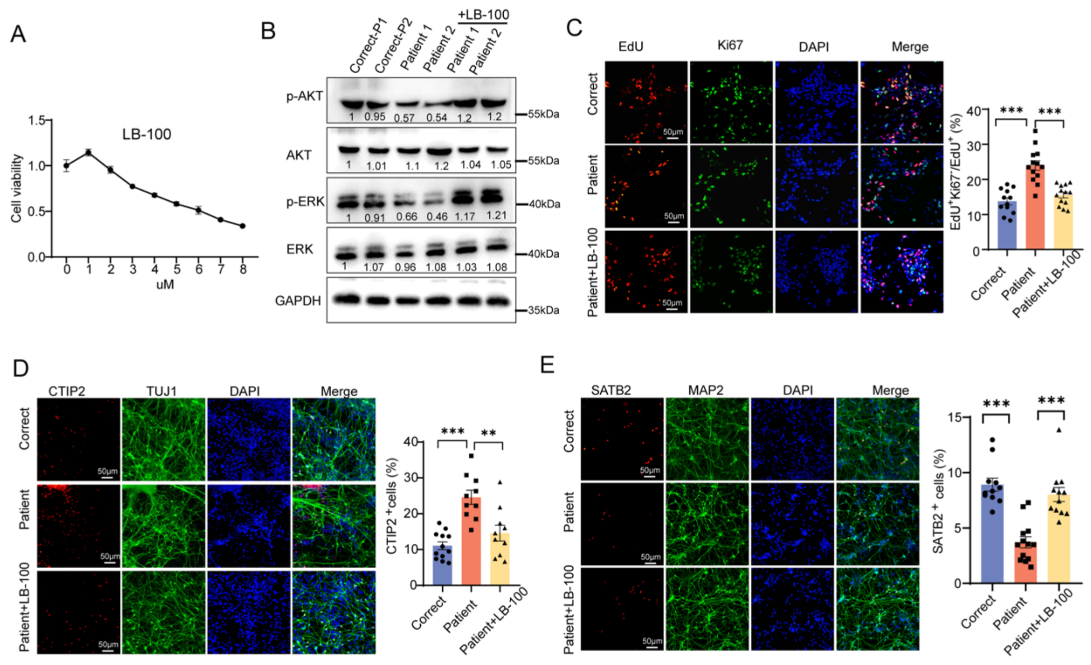

Supplementary Figure S6. Neurogenesis defects in the patient-derived NPCs are due to upregulated PP2A activity.

A) Cell viability determined by CCK8 assay. NPCs were treated with LB-100 at 1, 2, 3, 4, 5, 6, 7 and 8 μM for 24 h before detection. N=3. B) Western blots showing the expression of the indicated proteins in NPCs. The number below each band is the normalized ratio between the intensity of the band and the intensity of GAPDH band from the same sample. C) Ki67 staining (green) of the indicated NPCs at 48 h after EdU labeling (red). Scale bar, 50 μm. The bar graphs show the quantification of the percentage of EdU^{+} $Ki67^{-}$ in EdU^{+} cells. N=12. D) Representative images of TUJ-1 and CTIP2 staining in day-40 neural culture and the quantification of the percentage of $CTIP2^{+}$ cells. N=10-13. E) Representative images of MAP2 and SATB2 staining in day-60 neural culture and the quantification of the percentage of $SATB2^{+}$ cells. Scale bar, 50 μm. N=10-13. Data are presented as the mean ± SEM. The statistical

significance was determined using one-way ANOVA with Tukey test. *: $P< 0.05$; **: $P< 0.01$; ***: $P< 0.001$. ns: no significance.

Supplementary Table S1. iPSC lines used in this study

iPSC line	Sex	Age	Ethnicity	Diagnosis	Genotype	Source	Karyotype	Expression of pluripotency markers	Mycoplasma
Normal 1	Male	22 years old	Han Chinese	Healthy	Normal	PBMCs	Normal	+	-
Normal 2	Male	3 years old	Han Chinese	Healthy	Normal	PBMCs	Normal	+	-
Correct-P1	Male	15 years old	Han Chinese	N/A	Normal	PBMCs	Normal	+	-
Correct-P2	Male	11 months	Han Chinese	N/A	Normal	PBMCs	Normal	+	-
Patient 1	Male	15 years old	Han Chinese	XLMR	*CUL4B* c.1564 C>T	PBMCs	Normal	+	-
Patient 2	Male	11 months	Han Chinese	XLMR	*CUL4B* c.1007_1011del	PBMCs	Normal	+	-

Supplementary Table S2. Analysis of predicted off-target sites

**gRNA for CUL4B 1564 C>T site**			
**Top off-target sequences**	**Position**	**Primers (5' to 3') for sanger sequencing**	**Off-targets**
GaCCTTCAGCTGCAaAGAGCTGG	chr4: -2117811	TGGGTTGGTGTGACAGATTAGA/TGTCAGCTTTTCAGACGATAGG	None detected
aaCCTTCAGCTGCATAGAGaTGG	chr3: +134337692	GAGTAGCCGCTTCGTAGTGATT/CCAAGTGAGTCCTGAAAAGGTC	None detected
GaCCTcCAGCTGCAcAGAGCAGG	chr8: +27064851	AAAAGAGAGGCAGAGGACTTGA/CTGTTGATCTCTCAGGGTCTCA	None detected
aGCtTcCAGCTGCATAGAGCTGG	chr5: +132648887	AGCTCAGGTGTGTCTTTTCCTT/ACGAGAGCCTATGTCTGTAGCC	None detected
GtCCcTCAaCTGCATAGAGCAGG	chr1: +181934684	TCCCAAACCTCATTTCTCATCT/GGAGCTGAATGTTAATCACCAA	None detected
tGCCagCAGCTGCATAGAGCGGG	chr20: -35958954	GAGACAGGTGTTGGTGACAAGA/GCTTCCTCCTAATGCACTCAAG	None detected
GGCCTTCAGaTGACATAGAGCTGG	chr6: + 19051060	TGGTTCATGGGAAAAGGATTAG/CCTGGCTTAGAGGTCAGTTGAG	None detected
GGCtTTCAGGCTGtATAGAGCTGG	chr8: +36162235	AAAGGGCATTGATGTTGAAGTC/AGTAGGTGTTTCCGCTGAGAAG	None detected
**gRNA for CUL4B c.1007_1011del site**			
**Top off-target sequences**	**Position**	**Primers(5' to 3') for sanger sequencing**	**Off-targets**
GTGaAagATAAGACAATTGATGG	chr8: -75950999	GCACAACCACTTTGAAAAACAG/TACTGTCTTCTGCCTTGCCTTT	None detected
GTtCAGAAaAAGAgAATTGATGG	chr13: +103049764	GTTTTGGCACCTAAAACAGCTT/CACACAGGATATCCCCAAACTT	None detected
aTGCAGAATAcGACAAcTGATGG	chr8: +3770210	ATAACAGAGGGGGCTGGTTAAT/CCCTCCAGAACTCTCAACAGAA	None detected
GaGacGAATAAGACAATTGACAG	chr12: +128440384	TGTACCTCTACAGGACCAAAGAA/ACAGGACTGAGGATCAGAGAGG	None detected
GTGCAGAATAAGACACAaTGAAGG	chr2: -9194609	CACAGACTGAGTGGAGTTCACC/CAACCAAGGACAAAGAGTTTCC	None detected
GTGCAGCAAgAAGACAATTGATGG	chrX: -20928910	GGATAGGTGGAGGGATAGACAG/GAAAATTTCAAGCACGTGGA	None detected
tTGgtGAATgAGACAATTGAGGG	chr11: -66393894	GTAATCCAGCGAGGACTTTGTC/AAAATCCACCTTGGCTAGTTCA	None detected
GgGCAGATATAAGACAAaTGAGGG	chr9: -4939627	CTGGCACATTTTACCAGCAT/AGAGTTGGAAGGGACTTTAGGG	None detected

Supplementary Table S3. Primers and sequences

**qRT-PCR**		
	**Forward**	**Reverse**
CUL4B	ATGCTCCTCTGCCTGTTGAC	GCCGAATCCCTGGGTTGTAA
OCT4	CCTCACTTCACTGCACTGTA	CAGGTTTTCTTTCCCTAGCT
NANOG	AAGGTCCCGGTCAAGAAACAG	CTTCTGCGTCACACCATTGC
SOX2	CCCAGCAGACTTCACATGT	CCTCCCATTTCCCTCGTTTT
SOX1	GGCCGAGTGGAAGGTCATGT	GCCGGTACTTGTAATCCGGG
PAX6	TGGGCAGGTATTACGAGACTG	ACTCCCGCTTATACTGGGCTA
DCX	CCTTGGCTAGCAGCAACAGT	CCACTGCGGATGATGGTAA
TUBB3	GGCCAAGGGTCACTACACG	GCAGTCGCAGTTTTCACACTC
CUX1	GCTCTCATCGGCCAATCACT	TCTATGGCCTGCTCCACGT
MAP2	CTCAGCACCGCTAACAGAGG	CATTGGCGCTTCGGACAAG
FOXP2	AATGTGGGAGCCATACGAAG	GCCTGCCTTATGAGAGTTGC
TBR1	GCCTTTCTCCTTCTATCATGCTC	GTCAGTGGTCGAGATAATGGGA
CTIP2	TGGGTGCCTGCTATGACAAG	GGCTCGGACACTTTCCTGAG
SATB2	ACACCATCATCAAGTTCTTCCA	GCAGCTCCTCGTCCTTATATTC
PPP2R2B	CCGCTGATGACCTGAGGATTAACC	GTGGAACTCGGCTGCTGTGATC
PPP2R2C	TGGATCTGATGGTGGAGGTGAGC	TGTTGACGGAGATGGAGTTGATGTG
GAPDH	GTGGACCTGACCTGCCGTCT	GGAGGAGTGGGTGTCGCTGT

**sgRNA oligos**		
sgRNA#1 (correction c.1564C>T)	GGCCTTCAGCTGCATAGAGC	
sgRNA#2 (correction c.1564C>T)	CAAAAGAATCTTGATAAATC	
sgRNA#3 (correction c.1007_1011del)	GTGCAGAATAAGACAATTGA	
sgRNA#4 (correction c.1007_1011del)	CATGGGACTGGAGTTATTTA	
**HDR templates**		
ssODN#1 (correction c.1564C>T)	AAATAAATGATCTCCTTCATTTTGTTCCCCCTCCAGATTTATCAAGATTCTTTTGAACAACGATT TTTGGAAGAAACTAACCGcCTCTATGCAGCTGAAGGCCAAAAATTAATGCAAGAAA	
ssODN#2 (correction c.1007_1011del)	TTTAGGTTATATGTGTTTTTTATTTTAGGGACATGGGACTGGAGTTATTTCGCGCTCATATTATA AGTGATCAGAAAGTGCAGAATAAGACAATCGATGGCATTCTTCTCTTGATTGAGAGGGAAA	
**Sanger sequencing primers for genotype**		
	**Forward**	**Reverse**
CUL4B-1564	CCTACTATACAGAGCTGATTGCT	AAAAGATCCTGGAAGTGGCCC
CUL4B-1007	CAAACCTCTTCAGAAGTGGTATTG	CATTACCTGTCTGATGTGGGG
**siRNA sequences**		
	**Sense (5’-3’)**	**Antisense (5’-3’)**
si*PPP2R2B*	GGCGGCUACAAAUAACCUATT	UAGGUUAUUUGUAGCCGCCTT
si*PPP2R2C*	GCUCAUUCUUCUCGGAAAUTT	AUUUCCGAGAAGAAUGAGCTT
**ChIP-qPCR**		

	Forward	Reverse
PPP2R2B-6K	GCCAGTACCTACCAGCTTCAGT	AGTGATCCTTTAACGTGGCATT
PPP2R2B-5K	CAGACTGGCTTAGGTCAAAGGT	CAGCATGTGGATACATTCGTTT
PPP2R2B-4K	TATCCTTGGCACCTAGAGCAA	GGCAGGAGAATTGCTTGAAC
PPP2R2B-3K	TGAGGAAGTGGCAAAGAACA	TGTGGTGGAGGTAAGGGAGT
PPP2R2B-2K	TTAAGATCATTCCAACCGAAGG	CTATCACCGAGGTTCCAACATT
PPP2R2B-1K	GAGAGGCTGCAATAATTTGTCC	AGGCTGGTGTTCAATAAAGCAT
PPP2R2B+1K	GGGATCATGGATACTTGTCTGC	CGTTACCTTCGGTCGCATAG
PPP2R2C-6K	TGTGTGTGGTCAGCACTAATGA	CTTCCCAAACTCTACATCTGCAC
PPP2R2C-5K	CTGGTGATATGAACTGCTGCAC	TGTTTGTTCAGTGTGGAGGCTA
PPP2R2C-4K	TCCTGACTTGGTGGTTAGATGA	CTGTTGTTTACGCCACTTATCCT
PPP2R2C-3K	GAAAGTCATTCTCATGCCGTCT	GGTACAGCTACACTCGCCATCT
PPP2R2C-2K	AAGAAATCTGAGACGGCATCAC	CAGTGGAGGTAGGAGAGTCCAA
PPP2R2C-1K	CATGTACTTAATGCTCCCAACG	AGAAGGCAGAAAGGACTAGGAAG
PPP2R2C+1K	GGCTAATCTGGTGAAGAAATCTCG	GAGGATCTGGCAATGGATTATGTT

Supplementary Table S4. List of antibodies
Antibodies and their dilutions used for immunofluorescence staining.

**Antibody**	**Isotype**	**Dilution**	**Company**	**Cat.NO.**
NANOG	Rabbit IgG	1:200	Cell Signaling Technology	3580
SOX2	Rabbit IgG	1:400	Cell Signaling Technology	3579
SOX1	Rabbit IgG	1:200	Abcam	ab87775
SSEA4	Mouse IgG3	1:400	Cell Signaling Technology	4755
TRA-1-60	Mouse IgM	1:500	Cell Signaling Technology	4746
TRA-1-81	Mouse IgM	1:500	Cell Signaling Technology	4745
OCT4	Rabbit IgG	1:100	Proteintech	11263-1-AP
PAX6	Mouse IgG1	1:50	Abcam	ab78545
PAX6	Rabbit IgG	1:200	Proteintech	12323-1-AP
NESTIN	Mouse IgG1	1:100	Abcam	ab22035
CTIP2	Rat IgG2a	1:300	Abcam	ab8465
CTIP2	Rabbit IgG	1:200	Cell Signaling Technology	12120
CUX1	Mouse IgG3	1:50	Santa Cruz Biotechnology	sc-514008
TBR1	Rabbit IgG	1:400	Cell Signaling Technology	49661
TUJ1	Mouse IgG2a	1:400	Abcam	ab78078
MAP2	Mouse IgG1	1:400	Abcam	ab11268
TBR2	Rabbit IgG	1:200	Abcam	ab275960
SATB2	Rabbit	1:100	Abcam	ab92446
Synapsin I	Rabbit	1:1000	Merk	AB-1543
Ki67	Mouse IgG1	1:1000	Cell Signaling Technology	9449

N-cadherin	Mouse IgG1	1:50	Santa Cruz Biotechnology	sc-8424
Cleaved caspase-3	Rabbit IgG	1:400	Cell Signaling Technology	9664
DCX	Mouse IgG1	1:100	Santa Cruz Biotechnology	sc-271390
FOXG1	Rabbit	1:100	Abcam	ab196868
CUL4B	Rabbit	1:200	Sigma	HPA011880
Anti-Rabbit IgG Secondary Antibody, Alexa Fluor 488	Goat IgG	1:250	Thermo Fisher Scientific	A-11008
Anti-Mouse IgG Secondary Antibody, Alexa Fluor™ 488	Donkey IgG	1:250	Thermo Fisher Scientific	A-21202
Anti-Mouse IgG Secondary Antibody, Alexa Fluor™ 594	Donkey IgG	1:250	Thermo Fisher Scientific	A-21203
Anti-Rabbit IgG Secondary Antibody, Alexa Fluor™ 594	Donkey IgG	1:250	Thermo Fisher Scientific	A-21207
Anti-Rat IgG Secondary Antibody, Alexa Fluor™ 647	Goat IgG	1:500	Thermo Fisher Scientific	A-21247
Anti-mouse IgM Alexa Fluor 488	Goat	1:1000	Abcam	ab150121

Antibodies and their dilutions used for western blot.

Antibody	Isotype	Dilution	Company	Cat.NO.
p-AKT(S473)	Rabbit IgG	1:1000	Cell Signaling Technology	4060
AKT	Rabbit IgG	1:1000	Cell Signaling Technology	4691
p-ERK1/2 (T202/Y204)	Rabbit IgG	1:1000	Cell Signaling Technology	4370
ERK1/2	Rabbit IgG	1:1000	Cell Signaling Technology	4695
β-catenin	Mouse IgG1	1:1000	Santa Cruz Biotechnology	sc7963
p-GSK-3β (Ser9)	Rabbit IgG	1:1000	Cell Signaling Technology	9323
GSK-3β	Mouse IgG2a	1:1000	Abways Technology	AB3168
CyclinD1	Rabbit IgG	1:1000	Abways Technology	CY5404
p-CREB	Rabbit	1:1000	Cell Signaling Technology	9198
CREB	Rabbit IgG	1:1000	Cell Signaling Technology	9197
PPP2R2B	Mouse IgG1	1:1000	Proteintech	67783-1-Ig
PPP2R2C	Rabbit IgG	1:1000	Proteintech	12747-1-AP
GAPDH	Rabbit IgG	1:10000	Abways Technology	AB0037

Primary antibodies for ChIP

Antibody	Isotype	Company	Cat.NO.
H2AK119ub1	Rabbit	Millipore	ABE569
Histone H3 (trimethyl K27)	Mouse IgG3	Abcam	Ab6002
CUL4B	Rabbit	Sigma	C9995
Normal IgG	Rabbit	Cell Signaling Technology	2729
