# Supplementary material for: CUL4B mutations impair human cortical neurogenesis through PP2A-dependent inhibition of AKT and ERK
Source: Cell Death Dis. 2024 Feb 8;15(2):121. doi: 10.1038/s41419-024-06501-3 (PMC10853546; doi:10.1038/s41419-024-06501-3)

Full unedited blot/gel for Figure 1B

Figure 1B

Anti-CUL4B

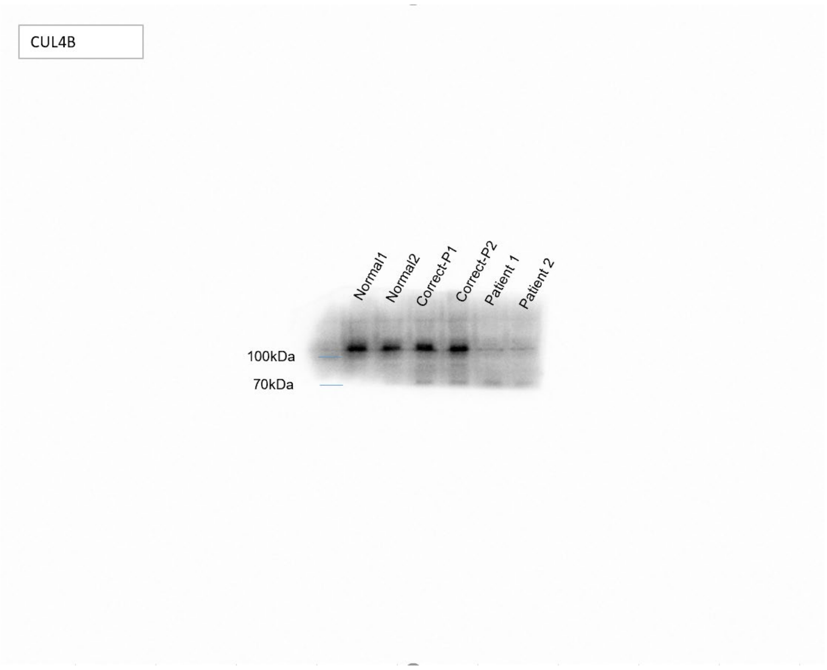

Anti-GAPDH

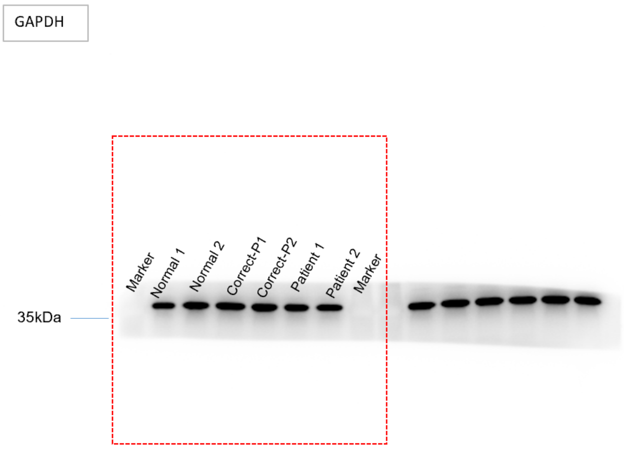

Full unedited blot/gel for Figure 5A

Figure 5A

Anti-GAPDH

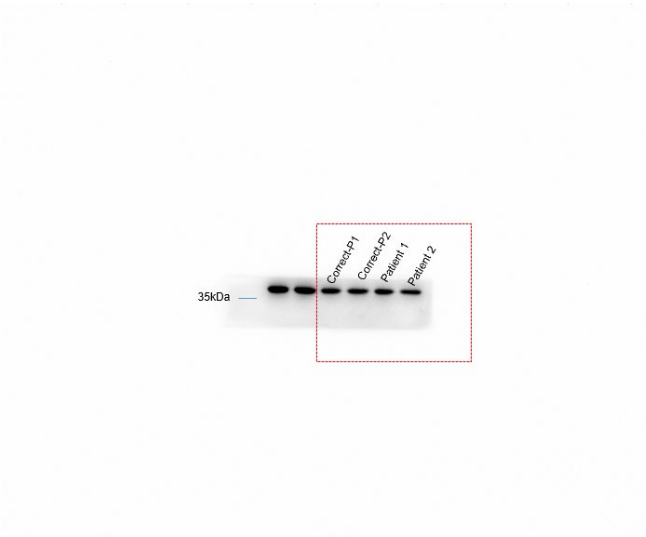

Anti-AKT

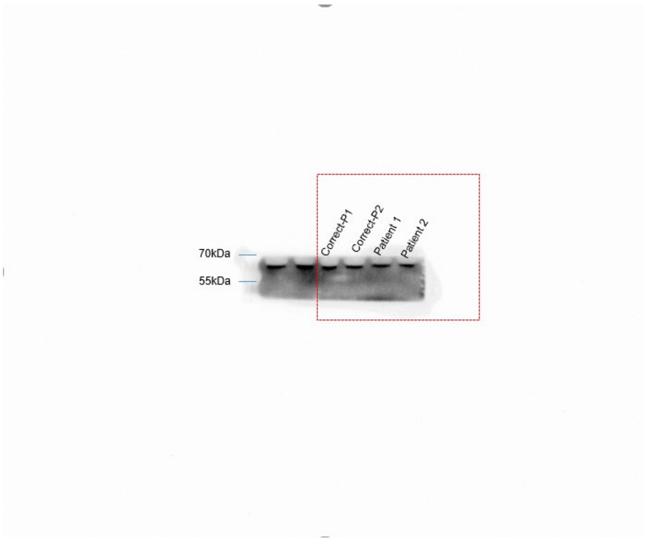

Anti-p-AKT

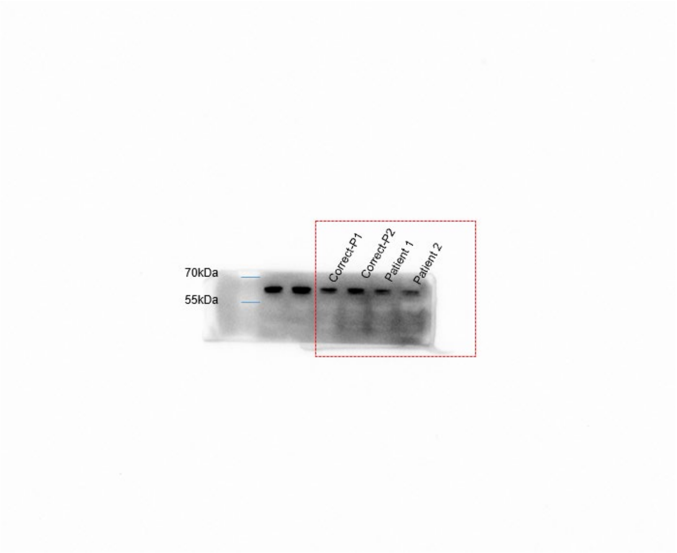

Anti-ERK

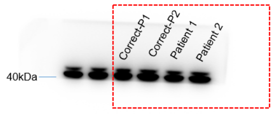

Anti-p-ERK

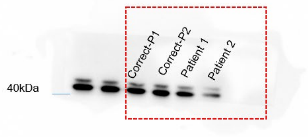

Anti-CUL4B

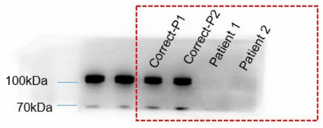

Anti-GAPDH

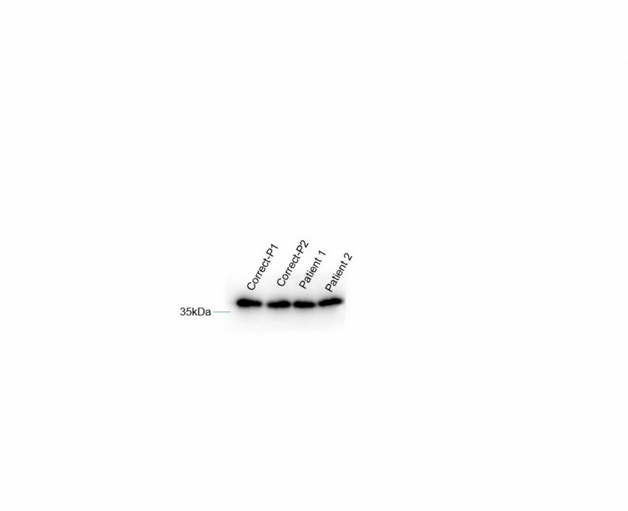

Anti-CREB

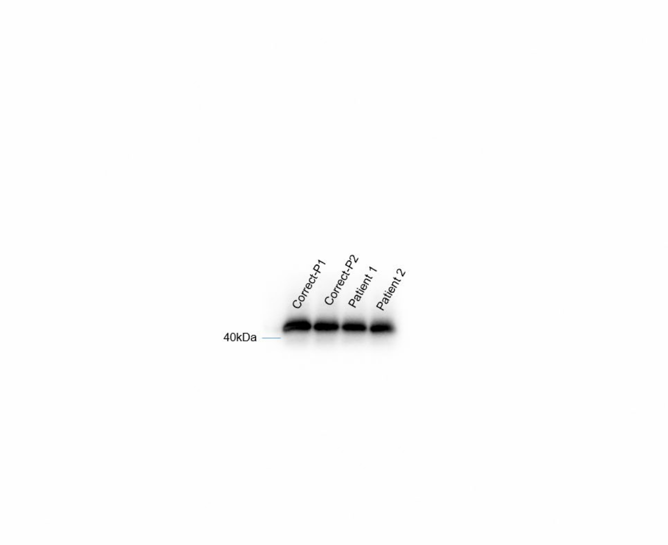

Anti-p-CREB

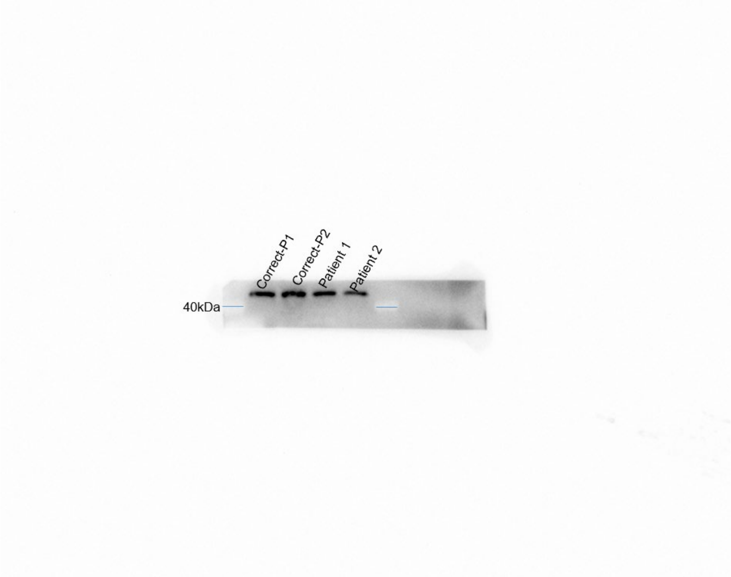

Anti-GSK3β

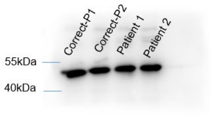

Anti-p- GSK3β

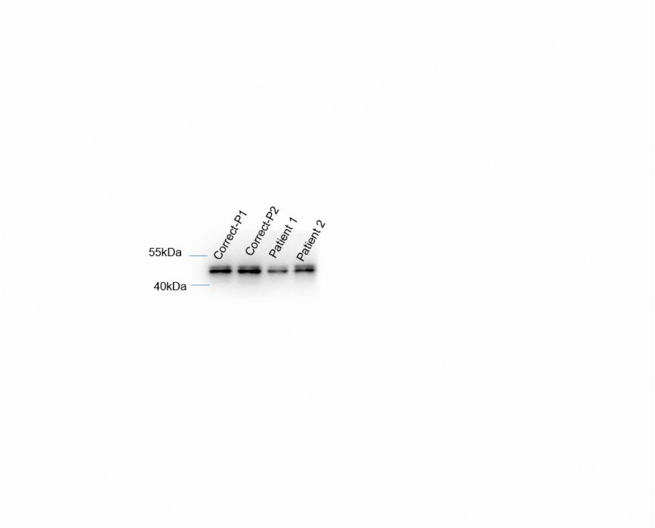

Anti-β-catenin

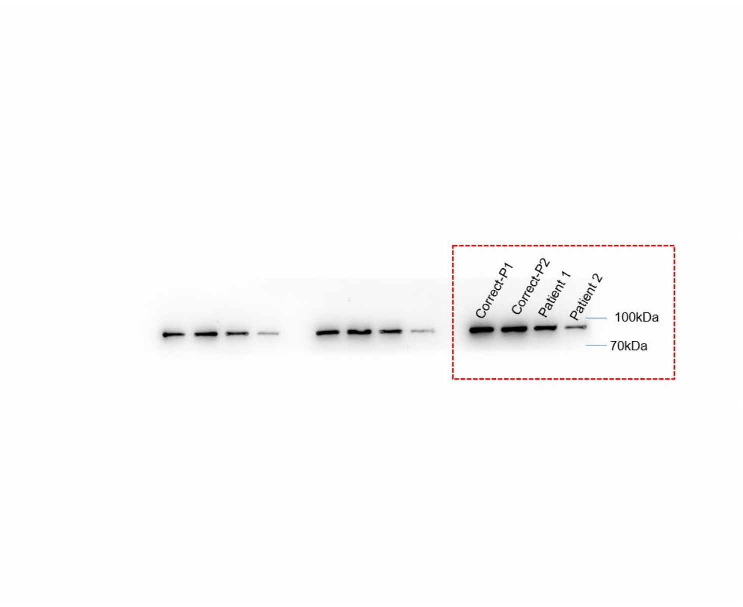

Anti-CyclinD1

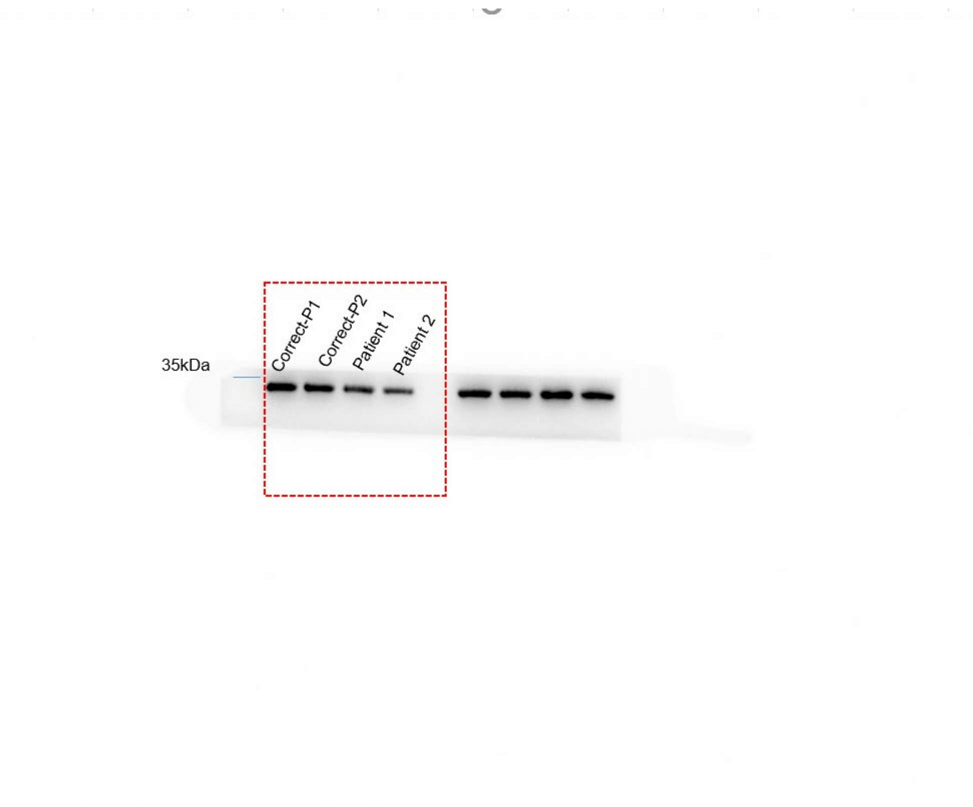

Full unedited blot/gel for Figure 5B

Figure 5B

Anti-GAPDH

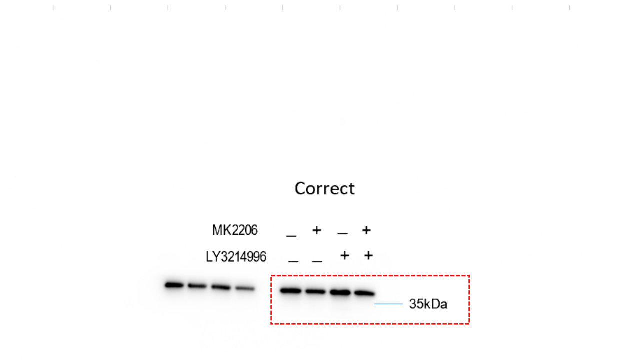

Anti-AKT

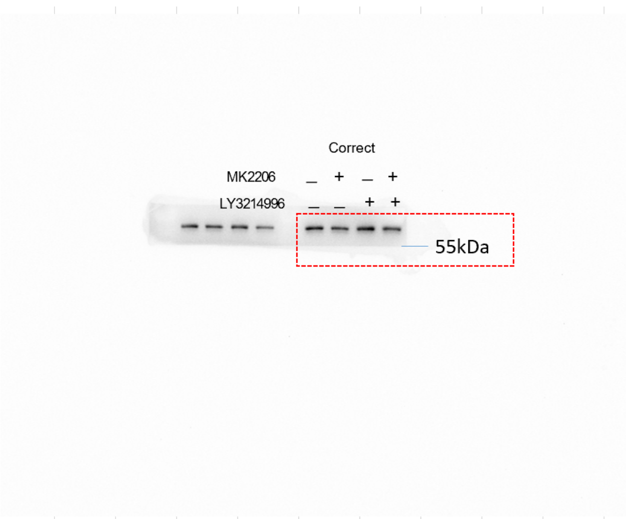

Anti-p-AKT

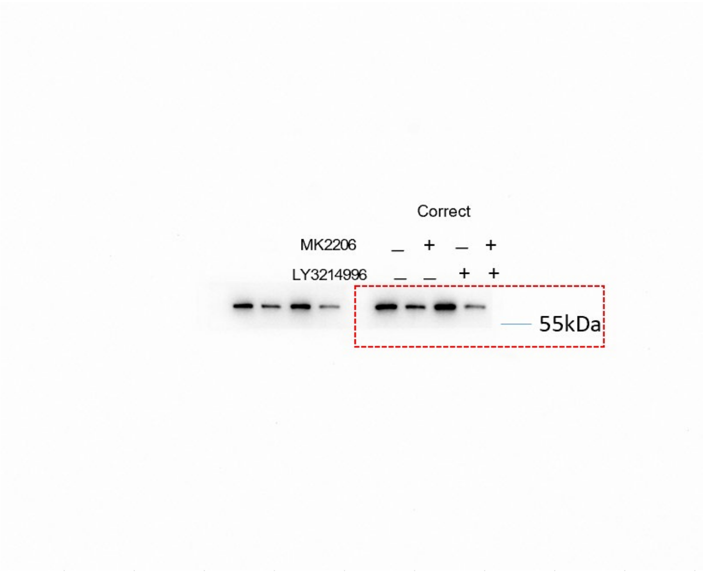

Anti-ERK

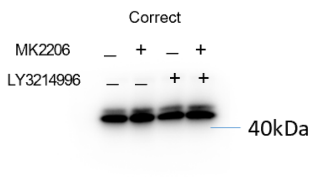

Anti-P-ERK

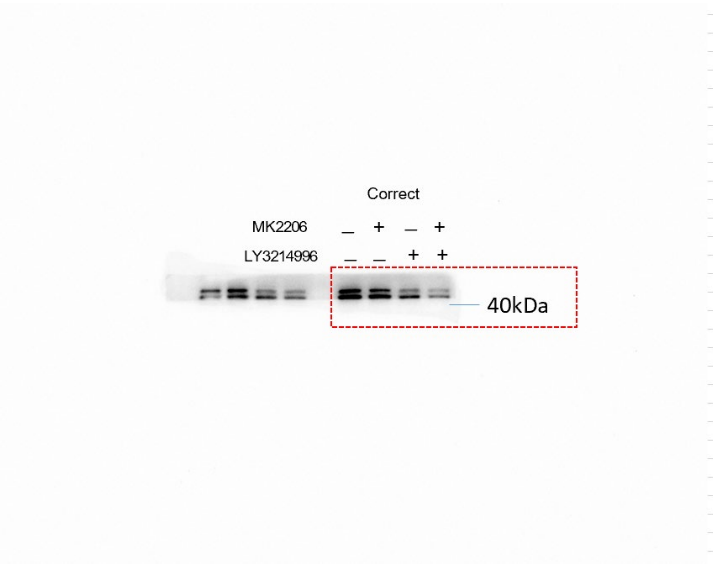

Anti-GAPDH

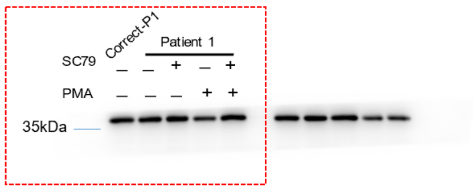

Anti-AKT

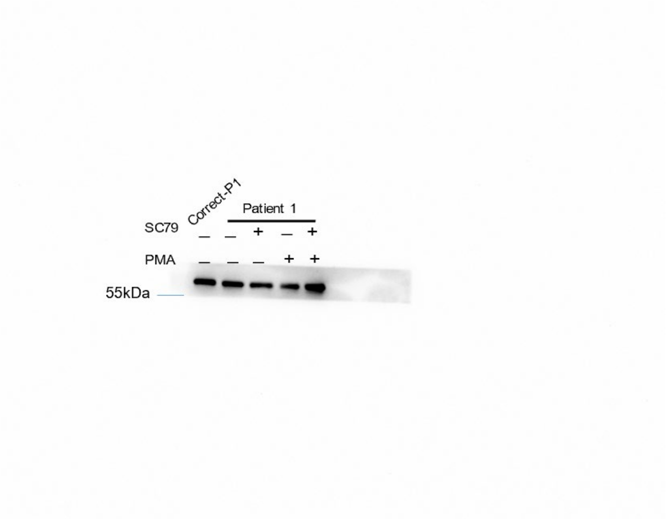

Anti-p-AKT

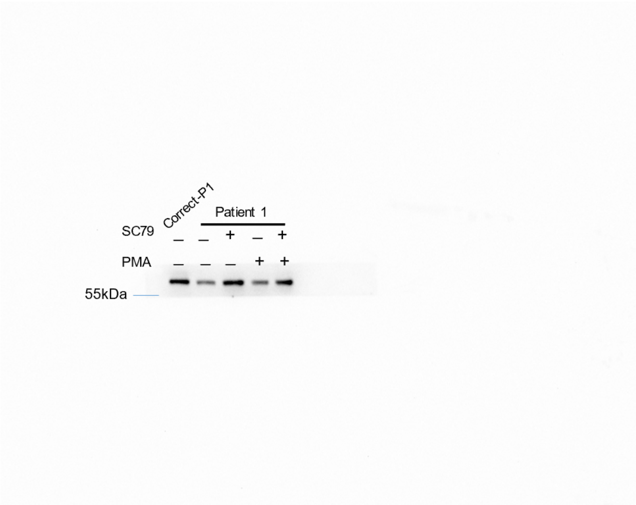

Anti-ERK

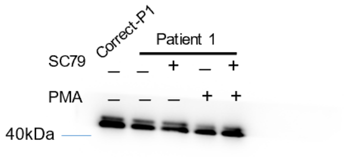

Anti-p-ERK

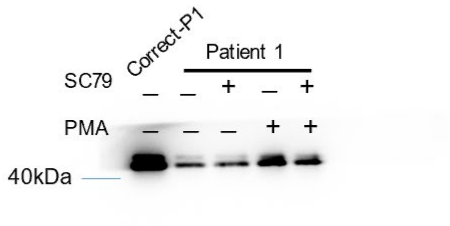

Full unedited blot/gel for Figure 6B

Figure 6B

Anti-GAPDH

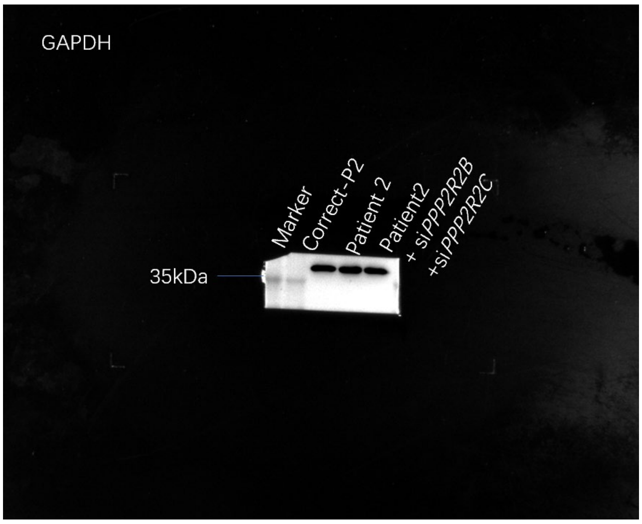

Anti-CUL4B

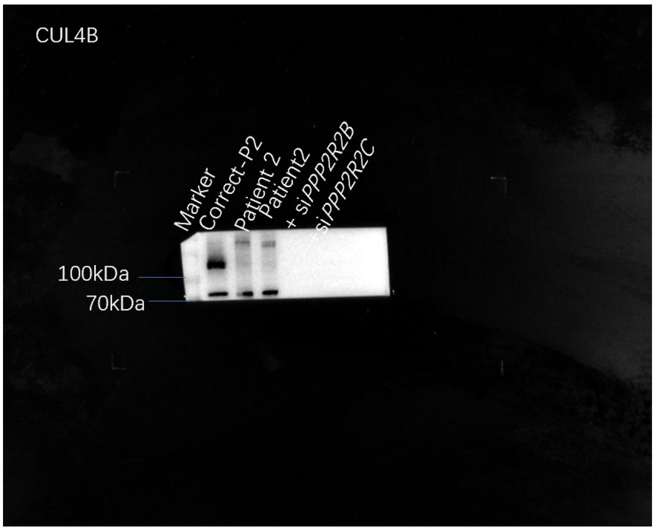

Anti-PPP2R2B

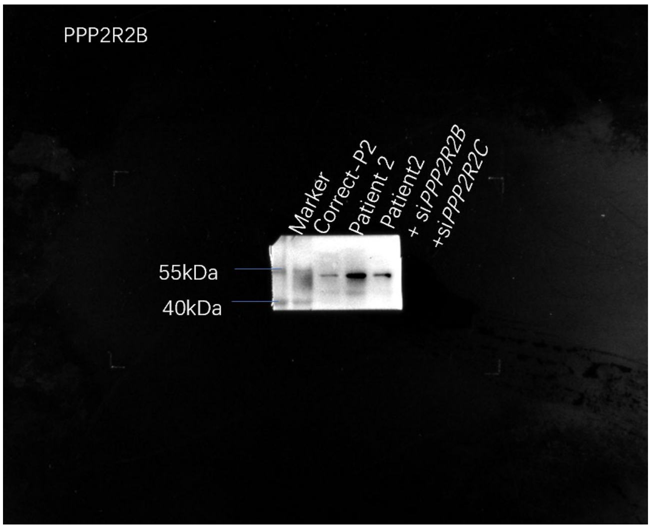

Anti-PPP2R2C

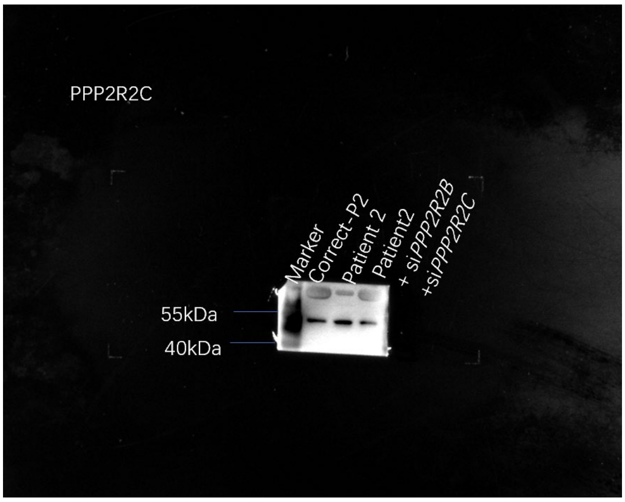

Anti-p-ERK

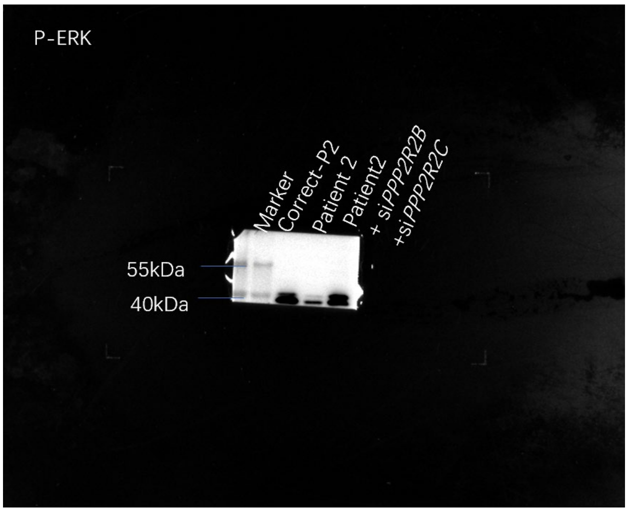

Anti-p-AKT

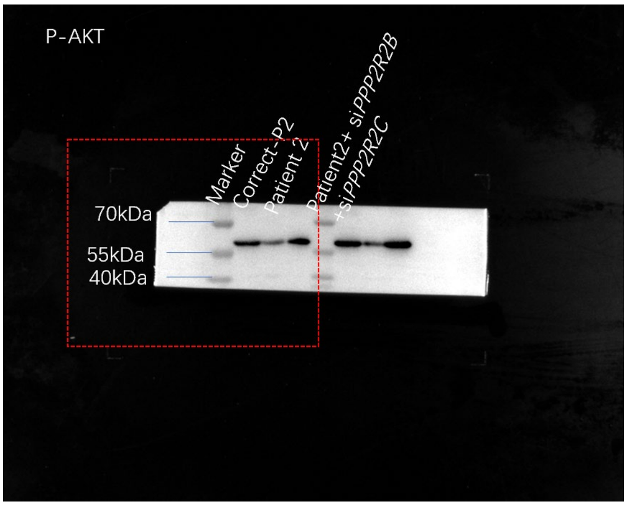

Full unedited blot/gel for Figure 6C

Figure 6C

Anti-GAPDH

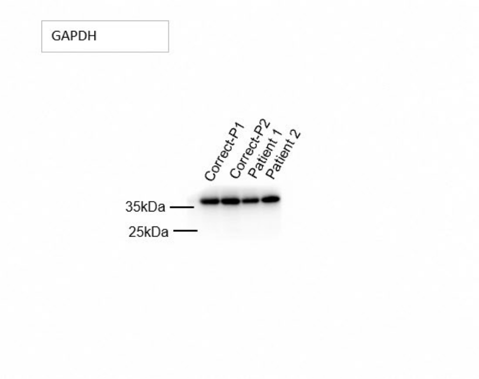

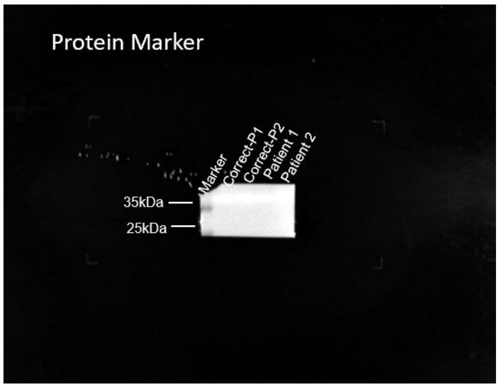

Anti-CUL4B

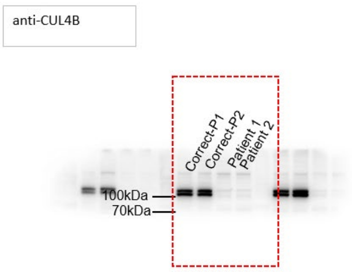

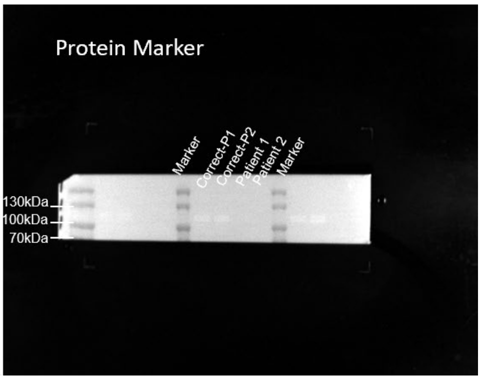

Anti-PPP2R2B

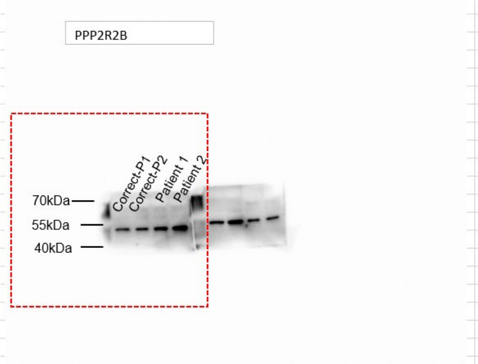

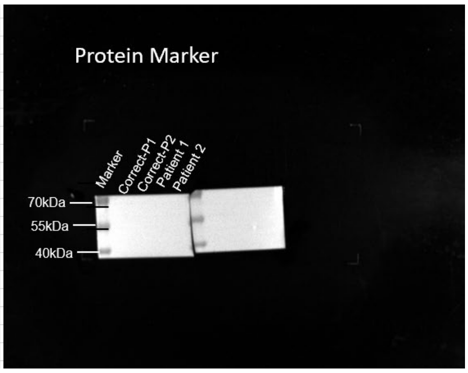

Anti-PPP2R2C

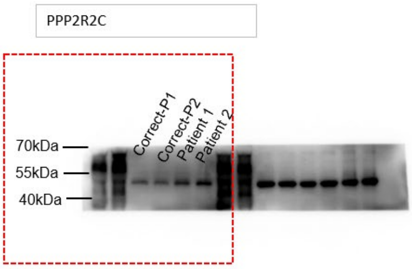

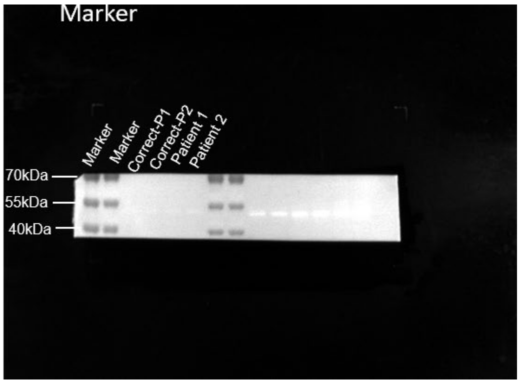

Full unedited blot/gel for Figure 6F

Figure 6F

Anti-GAPDH

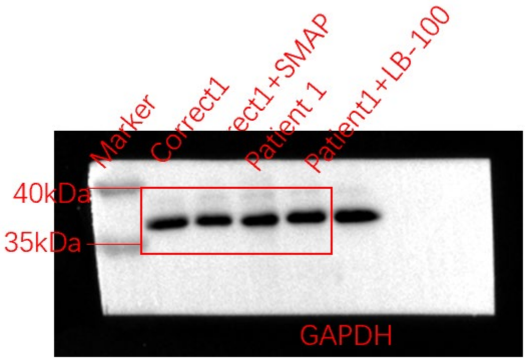

Anti-CUL4B

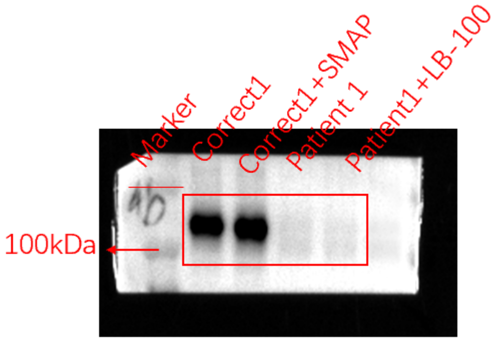

Anti-ERK

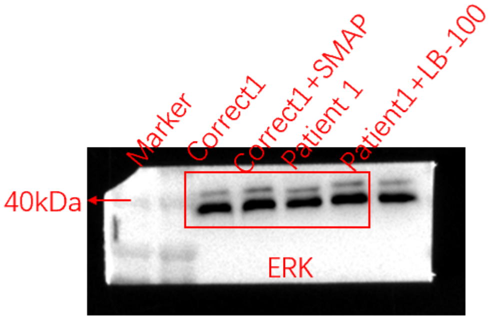

Anti-p-ERK

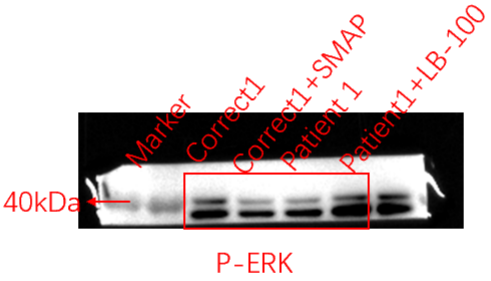

Anti-p-AKT

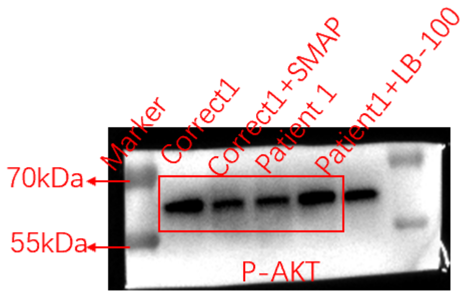

Anti-AKT

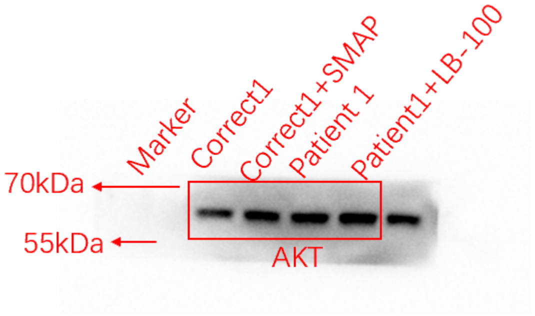

Full unedited blot/gel for Figure S2C

Figure S2C

CUL4B

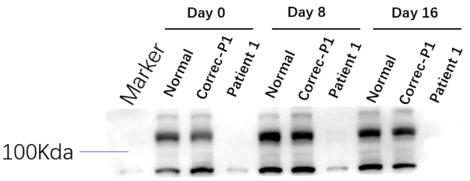

OCT4

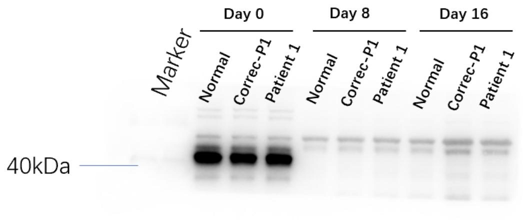

PAX6

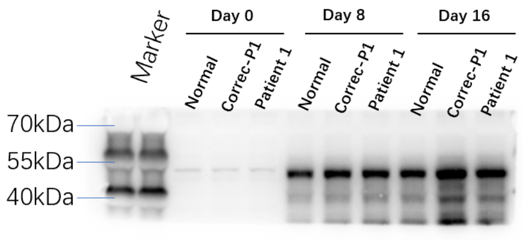

GAPDH

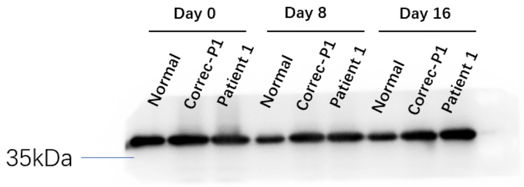

Full unedited blot/gel for Figure S4E

Figure S4E

Anti-GAPDH

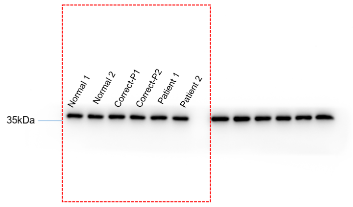

ERK

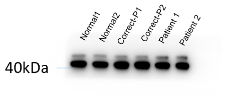

p-ERK

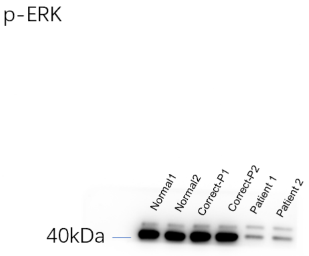

CUL4B

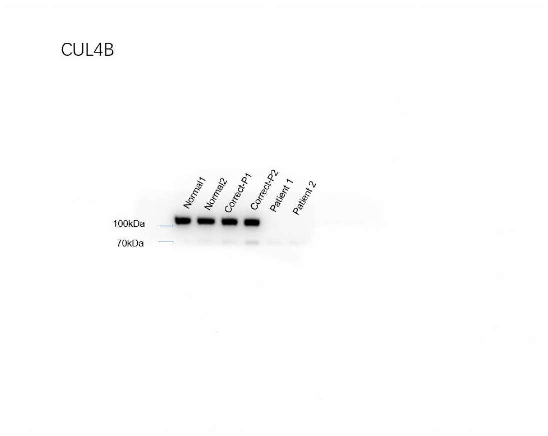

AKT

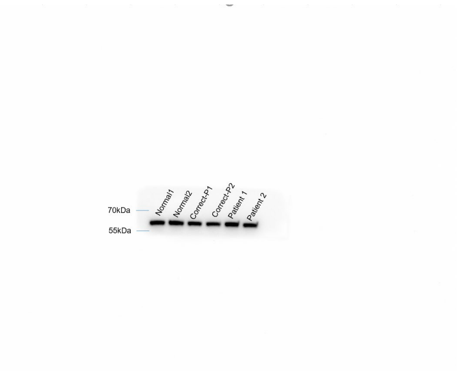

p-AKT

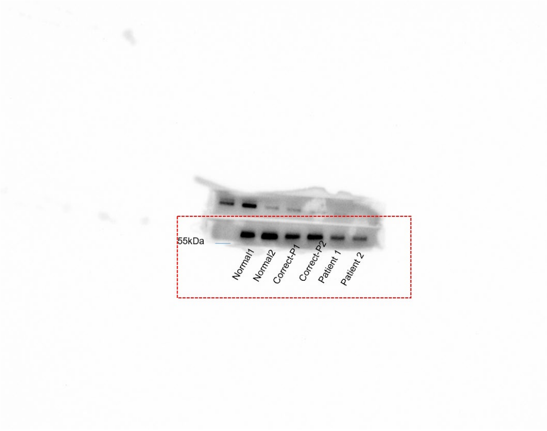

Anti-GAPDH

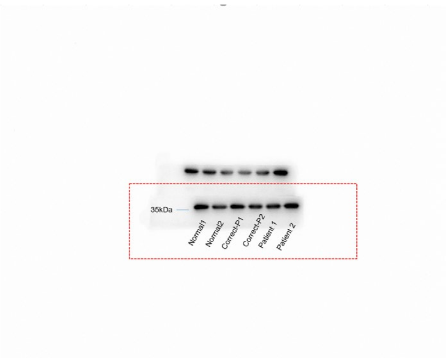

Anti -GSK3β

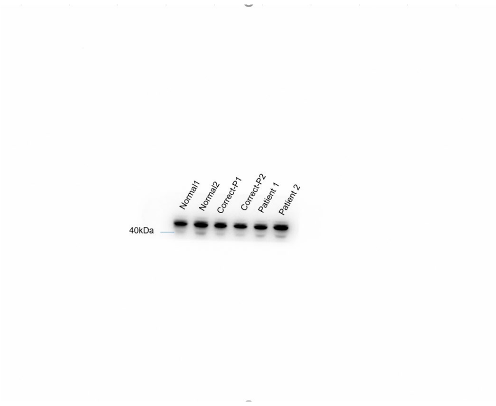

Anti-p-GSK3β

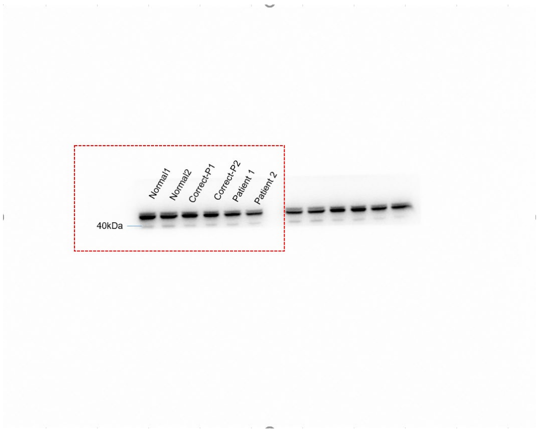

Anti-CREB

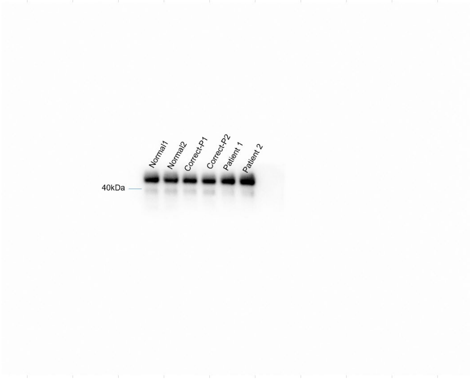

Anti-p-CREB

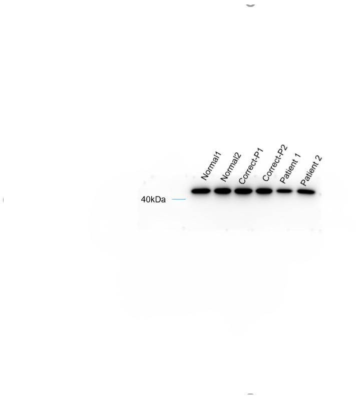

Anti-β-catenin

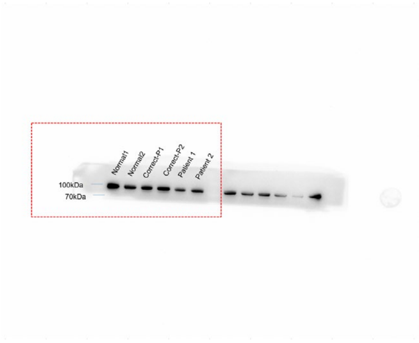

Anti-cyclinD1

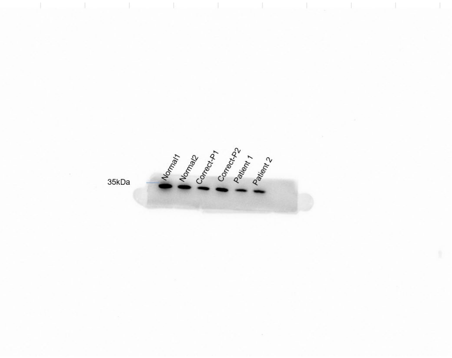

Full unedited blot/gel for Figure S6B

Figure S6B

Anti-GAPDH

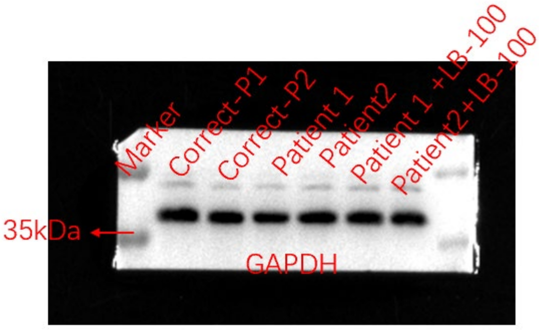

Anti-ERK

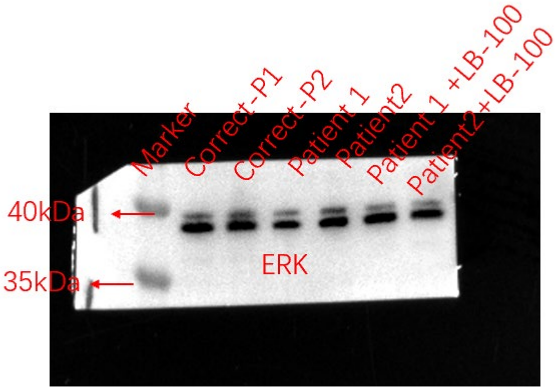

Anti-p-ERK

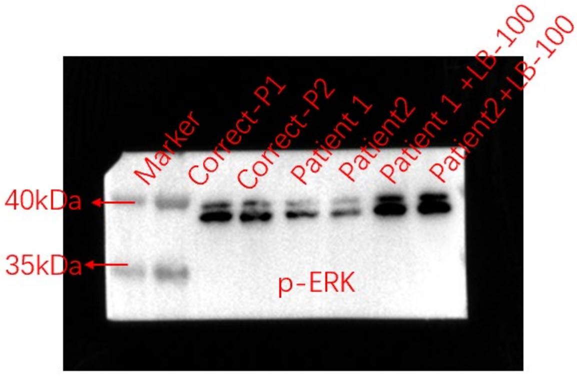

Anti-AKT

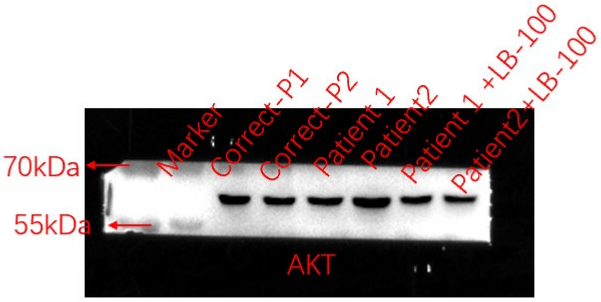

Anti-p-AKT

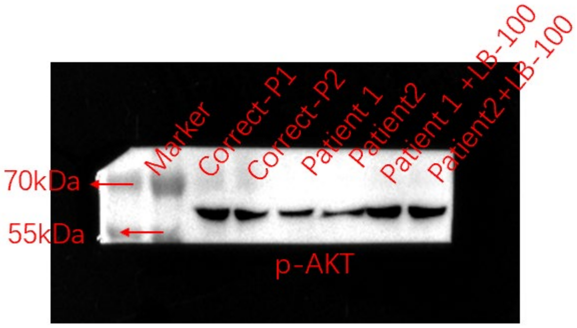

Supplement: Supplementary file 2 — Original Data File [file 41419_2024_6501_MOESM2_ESM.pdf]
